# Various short autonomously replicating sequences from the yeast *Kluyveromyces marxianus* seemingly without canonical consensus

**DOI:** 10.1016/j.crmicr.2021.100053

**Published:** 2021-07-31

**Authors:** Babiker M.A. Abdel-Banat, Hisashi Hoshida, Rinji Akada

**Affiliations:** a Date Palm Research Center of Excellence, King Faisal University, Al-Ahsa 31982, Saudi Arabia; b Department of Crop Protection, University of Khartoum, Shambat 13314, Sudan; c Department of Applied Molecular Bioscience, Yamaguchi University Graduate School of Medicine, Tokiwadai, Ube, Japan

**Keywords:** Functional validation, Interchanged sequences, *Km*ARS, NHEJ, Transformability

## Abstract

Eukaryotic autonomously replicating sequences (ARSs) are composed of three domains, A, B, and C. Domain A is comprised of an ARS consensus sequence (ACS), while the B domain has the DNA unwinding element and the C domain is important for DNA-protein interactions. In *Saccharomyces cerevisiae* and *Kluyveromyces lactis* ARS101, the ACS is commonly composed of 11 bp, 5ˊ-(A/T)AAA(C/T)ATAAA(A/T)-3ˊ. This core sequence is essential for *S. cerevisiae* and *K. lactis* ARS activity. In this study, we identified ARS-containing sequences from genomic libraries of the yeast *Kluyveromyces marxianus* DMKU3-1042 and validated their replication activities. The identified *K. marxianus* DMKU3-1042 ARSs (*Km*ARSs) have very effective replication ability but their sequences are divergent and share no common consensus. We have carried out point mutations, deletions, and base pairs substitutions within the sequences of some of the *Km*ARSs to identify the sequence(s) that influence the replication activity. Consensus sequences same as the 11 bp ACS of *S. cerevisiae* and *K. lactis* were not found in all minimum functional *Km*ARSs reported here except *Km*ARS7. Moreover, partial sequences from different *Km*ARSs are interchangeable among each other to retain the ARS activity. We have also specifically identified the essential nucleotides, which are indispensable for replication, within some of the *Km*ARSs. Our deletions analysis revealed that only 21 bp in *Km*ARS18 could retain the ARS activity. The identified *Km*ARSs in this study are unique compared to other yeasts’ ARSs, do not share common ACS, and are interchangeable.

## Highlights

• Identification of minimal autonomously replicating sequences (ARSs) from the yeast *Kluyveromyces marxianus* DMKU3-1042.
• The identities of the isolated ARSs are divergent and have no common consensus with the ARSs of other yeasts.
• A short ARS sequence of twenty-one nucleotides functions as an effective replicator in *K. marxianus* DMKU3-1042.
• Segments of ARSs from the yeast *K. marxianus* are interchangeable among each other.
• Functional ARSs are found in both the intergenic and coding sequences of the strain DMKU3-1042.

## 1 Introduction

Duplication of genomes requires precise initiation of DNA replication at replication origins. Eukaryotic replication origins are divergent but generally encompassed binding sites for origin recognition complex (ORC), regulatory sequences, and transcription units (Gilbert, 2001). An essential component of the replication origins is the *cis-*acting autonomously replicating sequence (ARS). ARS has been shown to allow stable maintenance of episomal plasmids within the yeast cell (Liachko and Dunham, 2014). Generally, intergenic sequences that contain more than 75% A-T are potential initiation sites for DNA replication in yeasts (Liachko et al., 2010). In *Saccharomyces cerevisiae*, short sequences less than 100 bp are defined as ARSs that contain 11-17 bp ARS consensus sequence (ACS) in addition to fairly defined flanking sequences (Liachko and Dunham, 2014; Méchali et al., 2013). However, Méchali (2010) reported that the presence of an ACS is not sufficient to predict a functional DNA replication origin because, among the 12,000 ACS sequences discovered in *S. cerevisiae* genomes, only 400 are active replicators (Nieduszynski et al., 2006). On the other hand, different groups within the genus *Saccharomyces* have varying ARS elements as components of the replication origin (Dhar et al., 2012). Most of *Kluyveromyces lactis* ARSs utilize 50 bp as an ACS motif, which is completely divergent from the canonical *S. cerevisiae* ACS (Liachko et al., 2010) except the ARS101 of *K. lactis* that shares the common ACS of *S. cerevisiae* (Irene et al., 2004). The yeast *Lachancea kluyveri* ARSs require a sequence that is similar but much longer than the ARS consensus sequence well defined in *S. cerevisiae* (Liachko et al., 2011). ARS elements in *Schizosaccharomyces pombe* are more than 1 kb in size, rich in AT residues, but lacking a common sequence motif. High-affinity binding of *S. pombe* ARS to *Sp*ORC requires no specific sequence (Clyne and Kelly, 1995; Kelly and Callegari, 2019; Reeves and Nissen, 1990). An ARS of 60 bp was reported as indispensable and adequate to confer ARS function to shuttle plasmids and linear DNAs in the yeast *Candida guilliermondii* (Foureau et al., 2013).

The yeast *Kluyveromyces marxianus* DMKU3-1042 is thermotolerant, fast growing on various carbon biomass, cost-effective, and high-temperature ethanol fermenting yeast (Abdel-Banat et al., 2010a; Limtong et al., 2007). It tends to effectively integrate linear DNA fragments randomly into its chromosomes (Nonklang et al., 2008) via its highly active non-homologous end-joining (NHEJ) pathway (Abdel-Banat et al., 2010b) and it does not need homology sequences at the fragment’ ends for effective recombination unless otherwise its NHEJ pathway is disrupted. To utilize the advantages of the strain, we developed a simple one-step method for NHEJ-based cloning and constructed several *K. marxianus* circular plasmids with different selection markers for recombinant DNA (Hoshida et al., 2014). Using this method, 36 promoters were cloned to express RFP, and promoters' activities and expression profiles were analyzed in a real-time manner (Suzuki et al., 2015). The outstanding notice is that transformation of a mixture of two PCR-amplified DNA fragments could generate correct recombinant DNA in *K. marxianus* and the replication of plasmids within the yeast cells was driven by the 60-bp sequence of *Km*ARS7 (Hoshida et al., 2014).

In this study, we demonstrate isolation and analysis of more *Km*ARSs from the yeast *K. marxianus* DMKU3-1042. Following a simple functional validation approach and post-transformation cellular events, we identified several robust *Km*ARSs. In addition, the impact of site-specific mutations and deletions on the activity of some *Km*ARSs were determined. We also demonstrate the influence of short interchanged sequences of *Km*ARSs on the replication activity. The *Km*ARSs reported here indicate that the strain DMKU3-1042 uses various autonomously replicating sequences that have no obvious canonical consensus.

## 2 Materials and methods

### 2.1 Strains, media, and transformation procedures

Yeast strains (Table 1) were regularly maintained at 28°C in YPD medium [1% yeast extract, 2% peptone, 2% glucose] or SD medium [0.17% yeast nitrogen base without amino acids and ammonium sulphate (US Biological, MA, USA), 0.5% ammonium sulphate, 2% glucose and required nutrients]. SD(–U) was an SD medium with necessary nutrients but lacking uracil (Ausubel et al., 1999). 5-Fluoroorotic acid (5-FOA) medium was prepared according to the protocol described by Akada et al. (2006). Luria–Bertani (LB) medium containing 100 μg/ml ampicillin (Sigma-Aldrich, MO, USA) was used for the selection of *E. coli* strain DH5α cells that transformed with plasmids bearing the Amp*^R^* marker gene. Solid media contained 2% agar. Yeast strains were grown in fresh YPD plates at 28°C for 1∼2 days before being used for transformation experiments. Yeast competent cells were prepared as previously described (Abdel-Banat et al., 2010b). Briefly, a mixture containing final concentrations of 40% w/v polyethylene glycol 3350 (PEG), 200 mM lithium acetate (LiAc), and 100 mM dithiothreitol (DTT) was dissolved in sterilized distilled water. This mixture was referred to as the transformation mixture (TM). Aliquots of auxotrophic mutant *K. marxianus* cell suspension prepared in the TM retain their competence for up to 14 months when stored at −80°C (Abdel-Banat et al., 2010b). The transformation was accomplished by thawing the yeast competent cells at room temperature, followed by the addition of PCR-amplified linear or plasmid DNA, heat shock for 15 min at 47°C, and then plating on SD(-U) medium for selection.

**Table 1.** Yeast strains used in this study.

Strain	Genotype	Parental strain	Reference
RAK3596	*K. marxianus* DMKU3-1042 wild type	-	Limtong et al. (2007)
RAK3605	*K. marxianus ura3-1*	RAK3596	Nonklang et al. (2008)
RAK3908	*K. marxianus ura3-1 ade2-1*	RAK3596	Hoshida et al. (2014)
RAK4174	*K. marxianus ura3 leu2*	RAK3605	Abdel-Banat et al. (2010b)
RAK4736	*K. marxianus ura3-1 leu2 ku70::*Sc*LEU2*	RAK4174	Abdel-Banat et al. (2010b)
BY4704	*S. cerevisiae MAT***a***ade2Δ::hisG his3Δ200 leu2Δ0 lys2Δ0 met15Δ0 trp1Δ63*	*S. cerevisiae MAT***a**	Brachmann et al. (1998)

### 2.2 Screening and isolation of autonomously replicating sequences from *K. marxianus* (*Km*ARSs)

The yeast *K. marxianus* DMKU3-1042 chromosomal DNA and the yeast *S. cerevisiae* shuttle vector pRS316 (Sikorski and Hieter, 1989) were digested with *Eco*RI and *Xho*I restriction enzymes as instructed by the manufacturer (New England Biolabs, MA, USA). The recovered *K. marxianus* DNA was ligated into the digested vector using the T4 DNA ligase kit (New England Biolabs, MA, USA) and the reaction was terminated by heating for 10 min at 65°C. The ligation product was transformed into competent cells of *E. coli*. Approximately 14,959 *E. coli* colonies carrying plasmids with *K. marxianus* chromosomal DNA fragments were pooled from the LB selection plates, cultured overnight in liquid LB medium at 37°C and the recombinant plasmids were extracted and purified from *E. coli* cells using QIAprep® spin miniprep kit (Qiagen). The purified plasmids were transformed again into the *K. marxianus* strain RAK3605 (*ura3-1*) as described previously (Abdel-Banat et al., 2010b). RAK3605 cells that were transformed with the genomic library were cultured in MM(−U) medium to identify the cells that harbor recombinant pRS316 with potential autonomously replicating sequences of *K. marxianus* (*Km*ARSs). The recovered cells were spread on YPD plates to produce colonies and subsequently, at least six transformants from each construct were inoculated on 5-FOA plates (Boeke et al., 1987) to detect whether these plasmids can replicate autonomously.

### 2.3 Sequence identification of *Km*ARSs

To identify the sequence of *Km*ARS-containing plasmids that confirmed replicating autonomously within *K. marxianus* cells, yeast transformants were cultured individually on MM(−U) liquid media and grown overnight at 28°C. Then plasmids were extracted using a Zymoprep^TM^ Yeast Plasmid Miniprep Kit II (Zymo Research, Orange, CA, USA) and Zymolyase 100 T (Seikagaku Biobusiness, Tokyo, Japan), as previously reported (Nonklang et al., 2008). Again, the isolated plasmids were cloned in *E. coli* DH5α competent cells and purified as stated in section 2.2. Throughout the empirical work in this study, the concentration of all kinds of DNA was quantified by Qubit® fluorometer (Thermo Fisher Scientific Inc.) using Quant-iT^TM^ dsDNA assay kit. The sequences of *Km*ARSs were determined by the cycle sequencing protocols used for the BigDye® Terminator v3.1 Cycle Sequencing Kit (Applied Biosystems™) according to the supplier's instructions. Recombinant pRS316 plasmids with inserted *Km*ARSs are listed in Table 2.

**Table 2:** Recombinant plasmids with *K. marxianus* genomic sequence and *Km*ARS characteristics.

Plasmid*	*Km*ARS size (bp)	Minimum functional ARS (bp)	Chromosomal site**	ACS***	GenBank® accession number
pRS316+*Km*ARS7	789	Nucleotides 201∼247: (47)	Chr 3: 1026687∼1027475	**143**-TTTTATATTTT-**153**	AB861609.1
pRS316+*Km*ARS11	154	Nucleotides 46~95: (50)	Chr 1: 1488492~1488645	-	MZ514902
pRS316+*Km*ARS16	1187	Nuleotides 721~770 (50)	Chr 3: 1557209~1556023	-	MZ514892
pRS316+*Km*ARS18	1349	Nucleotides 116~136: (21)	Chr 3: 861517~860169	-	MZ514893
pRS316+*Km*ARS22	1175	Nucleotides 1001~1050: (50)	Chr 6: 728944~730118	-	MZ514894
pRS316+*Km*ARS36	1183	Nucleotides 291~328: (38)	Chr 2: 770606~769424	-	MZ514895
pRS316+*Km*ARS51	795	Nucleotides 491~550: (60)	Chr 3: 358583~357789	-	MZ514896
pRS316+*Km*ARS14	1038	NI	Chr 7: 752365~753402	-	MZ514897
pRS316+*Km*ARS45	568	NI	Chr 4: 1411986~1412553	-	MZ514898
pRS316+*Km*ARS3	2590	NI	Chr 5: 839614~842203	-	MZ514899
pRS316+*Km*ARS20F	906	NI	Chr 6: 265137~266042	-	MZ514900
pRS316+*Km*ARS20R	905	NI	Chr 2:949414~950318	-	MZ514901

*pRS316 is a *S. cerevisiae* CEN6/ARSH4 shuttle vector (Sikorski and Hieter, 1989).

**Sequence coordinates represent the chromosomes of *K. marxianus* DMKU3-1042 (Lertwattanasakul et al., 2015).

***ACS, ARS consensus sequence commonly found in *S. cerevisiae* and *K. lactis* [(A/T)TTTAT(A/G)TTT(A/T)].

NI, Not Investigated.

### 2.4 DNA manipulation

PCR was performed using KOD plus DNA polymerase (Toyobo, Osaka, Japan) according to the manufacturer's instructions. The primers used are listed in Table 3. The *S. cerevisiae URA3* gene (*ScURA3*), including its promoter and terminator, was amplified by PCR from BY4704 chromosomal DNA with the following primer pairs: URA3-223 and URA3-300c; URA3-300 and URA3-300c; 9C-URA3-223 and URA3-300c; and 9C-URA3-223 and 3CG9-URA3+880c. The 9C and 3CG9 sequences flanking the *URA3* gene were utilized subsequently in two discrete PCR reactions (Cha-aim et al., 2009, 2012; Hoshida et al., 2014) to anneal the *Km*ARSs at either or both ends for further analysis. The minimum active sequences of *Km*ARSs (Table 2) were determined empirically by PCR-directed deletion of the *Km*ARS sequences from both sides and rejoining the amplified fragments together with the *URA3* gene as described before (Hoshida et al., 2014).

**Table 3:** Primers used in this study.

Primer name	Sequences (5′→3′)
URA3-223	AAGCTTTTCAATTCATCTTTTTTTTTTTTG
9C-URA3-223	cccccccccAAGCTTTTCAATTCATCTTTTTTTTTTTTG
URA3-300c	TGTTGTGAAGTCATTGACACAG
3CG9-URA3+880c	cccgggcccGTAATAACTGATATAATTAAATTGA
KmARS7(201-250)9c	CAAGACTTCTTGAAGTGAAAACCAACTTTCAGTCTTCAAACTAAAAATGAccccccccc
KmARS7(216-250)9c	GAAAACCAACTTTCAGTCTTCAAACTAAAAATGAAccccccccc
KmARS7(226-250)9c	CTTTCAGTCTTCAAACTAAAAATGAccccccccc
KmARS7(225-250)9C	ACTTTCAGTCTTCAAACTAAAAATGAccccccccc
KmARS7(219-250)9c	AACCAACTTTCAGTCTTCAAACTAAAAATGAccccccccc
KmARS7(222-250)9c	CCAACTTTCAGTCTTCAAACTAAAAATGAccccccccc
KmARS7(225-250)9c	CTTTCAGTCTTCAAACTAAAAATGAccccccccc
KmARS7(230-250)9C	CAGTCTTCAAACTAAAAATGAccccccccc
KmARS7(226-247)9c	CTTTCAGTCTTCAAACTAAAAAccccccccc
KmARS7(226-244)9c	CTTTCAGTCTTCAAACTAAccccccccc
KmARS7(226-241)9c	CTTTCAGTCTTCAAACccccccccc
KmARS7(226-238)9c	CTTTCAGTCTTCAccccccccc
KmARS7(226-235)9c	CTTTCAGTCTccccccccc
KmARS7(201-218)c-3CG9	TCACTTCAAGAAGTCTTGcccgggccc
KmARS7(201-221)c-3CG9	TTTTCACTTCAAGAAGTCTTGcccgggccc
KmARS7(201-224)c-3CG9	TGGTTTTCACTTCAAGAAGTCTTGcccgggccc
KmARS7(201-225)c-3CG9	TTGGTTTTCACTTCAAGAAGTCTTGcccgggccc
KmARS7(201-229)c-3CG9	AAAGTTGGTTTTCACTTCAAGAAGTCTTGcccgggccc
KmARS11(46-105)10c	TCCAAAATTAACTTTCTAAGCTAAATGTCATATTTCGCAATAAAATAATAAGAATATAGAcccccccccc
KmARS11(46-60)c-3CG9	AAAGTTAATTTTGGAcccgggccc
KmARS11(61-100)9C	CTAAGCTAAATGTCATATTTCGCAATAAAATAATAAGAATcccccccccc
KmARS11(46-100)9c	TCCAAAATTAACTTTCTAAGCTAAATGTCATATTTCGCAATAAAATAATAAGAATccccccccc
KmARS11(46-95)9c	TCCAAAATTAACTTTCTAAGCTAAATGTCATATTTCGCAATAAAATAATAccccccccc
KmARS11(76-105)9C	TATTTCGCAATAAAATAATAAGAATATAGAccccccccc
KmARS11(46-75)c3CG9	TGACATTTAGCTTAGAAAGTTAATTTTGGAcccgggccc
KmARS11(26-105)10c	CACTTTTTACACTGTGACGTTCCAAAATTAACTTTCTAAGCTAAATGTCATATTTCGCAATAAAATAATAAGAATATAGAcccccccccc
KmARS11(11-90)10c	AATCAATGATTCATACACTTTTTACACTGTGACGTTCCAAAATTAACTTTCTAAGCTAAATGTCATATTTCGCAATAAAAcccccccccc
KmARS11(36-105)10c	ACTGTGACGTTCCAAAATTAACTTTCTAAGCTAAATGTCATATTTCGCAATAAAATAATAAGAATATAGAcccccccccc
KmARS11(46-105)10c	TCCAAAATTAACTTTCTAAGCTAAATGTCATATTTCGCAATAAAATAATAAGAATATAGAcccccccccc
KmARS11(56-105)10c	ACTTTCTAAGCTAAATGTCATATTTCGCAATAAAATAATAAGAATATAGAcccccccccc
KmARS11(51-120)10c	AATTAACTTTCTAAGCTAAATGTCATATTTCGCAATAAAATAATAAGAATATAGATATCAAAGGTCTGTGcccccccccc
KmARS16(721-780)9c	TTTTATTTTTTTTTAACTCAATTTCCAGTTTAAACACCAAAATACGTTTCCATATAATTGccccccccc
KmARS16(721-770)9c	TTTTATTTTTTTTTAACTCAATTTCCAGTTTAAACACCAAAATACGTTTCccccccccc
KmARS16(731-790)10c	TTTTAACTCAATTTCCAGTTTAAACACCAAAATACGTTTCCATATAATTGAAAAAGGAAGcccccccccc
KmARS16(741-790)10c	ATTTCCAGTTTAAACACCAAAATACGTTTCCATATAATTGAAAAAGGAAGcccccccccc
KmARS16(751-790)10c	TAAACACCAAAATACGTTTCCATATAATTGAAAAAGGAAGcccccccccc
KmARS16(721-752)c-3CG9	AAAATACGTTTCCATATAATTGAAAAAGGAAGcccgggccc
KmARS16(753-790)9C	GGTGTTTAAACTGGAAATTGAGTTAAAAAAAAATAAAAccccccccc
KmARS16(731-790)10c	TTTTAACTCAATTTCCAGTTTAAACACCAAAATACGTTTCCATATAATTGAAAAAGGAAGcccccccccc
KmARS16(741-790)10c	ATTTCCAGTTTAAACACCAAAATACGTTTCCATATAATTGAAAAAGGAAGcccccccccc
KmARS16(751-790)10c	TAAACACCAAAATACGTTTCCATATAATTGAAAAAGGAAGcccccccccc
KmARS16(721-790)10c	TTTTATTTTTTTTTAACTCAATTTCCAGTTTAAACACCAAAATACGTTTCCATATAATTGAAAAAGGAAGcccccccccc
KmARS18(111-159)10C	TCCATAATTTGGAATTGAAAGTCACTTTAGGTTCACTATATAATGAAAAcccccccccc
KmARS18(111-149)c-3cG9	ATAGTGAACCTAAAGTGACTTTCAATTCCAAATTATGGAcccgggccc
KmARS18(121-149)9C	GGAATTGAAAGTCACTTTAGGTTCACTATATAATGAAAAccccccccc
KmARS18(111-138)c-3CG9	AAAGTGACTTTCAATTCCAAATTATGGAcccgggccc
KmARS18(139-159)9C	AGGTTCACTATATAATGAAAAccccccccc
KmARS18(111-134)c-3CG9	TGACTTTCAATTCCAAATTATGGAcccgggccc
KmARS18(135-160)9C	CTTTAGGTTCACTATATAATGAAAAGccccccccc
KmARS18(135-156)9C	CTTTAGGTTCACTATATAATGAccccccccc
KmARS18(135-153)9C	CTTTAGGTTCACTATATAAccccccccc
KmARS18(135-150)9C	CTTTAGGTTCACTATAccccccccc
KmARS18(135-147)9C	CTTTAGGTTCACTccccccccc
KmARS18(135-144)9C	CTTTAGGTTCccccccccc
KmARS18(135-141)9C	CTTTAGGccccccccc
KmARS18(135-138)9C	CTTTccccccccc
KmARS22(991-1060)9c	TGTTATCTTTTTCGCTTCAAAAGTTACTTTGGATTCTAATATAAGAAAAAAAATAAAAACAAACCAAATCccccccccc
KmARS22(1001-1060)9c	TTCGCTTCAAAAGTTACTTTGGATTCTAATATAAGAAAAAAAATAAAAACAAACCAAATCccccccccc
KmARS22(1001-1050)9c	TTCGCTTCAAAAGTTACTTTGGATTCTAATATAAGAAAAAAAATAAAAACccccccccc
KmARS22(1001-1020)c-3CG9	AAAGTAACTTTTGAAGCGAAcccgggccc
KmARS22(1021-1050)9C	GGATTCAAATATAAGAAAAAAAATAAAAACccccccccc
KmARS36(291-340)10c	TCTTTAATATTATTTTTCATTTCAAAAAGTGTGAAATAAAAATTAAAATGcccccccccc
KmARS36(291-306)c-3CG9	AAAATAATATTAAAGAcccgggccc
KmARS36(307-340)9C	TCATTTCAAAAAGTGTGAAATAAAAATTAAAATGccccccccc
KmARS36(307-337)9C	TCATTTCAAAAAGTGTGAAATAAAAATTAAAccccccccc
KmARS36(307-334)9C	TCATTTCAAAAAGTGTGAAATAAAAATTccccccccc
KmARS36(307-331)9C	TCATTTCAAAAAGTGTGAAATAAAAccccccccc
KmARS36(307-328)9C	TCATTTCAAAAAGTGTGAAATAccccccccc
KmARS36(307-325)9C	TCATTTCAAAAAGTGTGAAccccccccc
KmARS36(307-322)9C	TCATTTCAAAAAGTGTccccccccc
KmARS36(316-340)9C	AAAGTGTGAAATAAAAATTAAAATGccccccccc
KmARS36(291-315)c3CG9	TTGAAATGAAAAATAATATTAAAGAcccgggccc
KmARS51(491-550)9c	AATATTTATGAATAAAAGTAACTTTTTAGTTTCAAATACTAAAAAATATTAATTACAAAGccccccccc
KmARS51(491-515)c-3CG9	AAAGTTACTTTTATTCATAAATATTcccgggccc
KmARS51(516-550)9C	TTAGTTTCAAATACTAAAAAATATTAATTACAAAGccccccccc

### 2.5 Functional validation of *K. marxianus* ARSs (*Km*ARSs) by linear *Km*ARS transformation

To determine the minimum active sequences of *Km*ARSs, three steps were followed (Fig. S1A). First, the *ScURA3* gene was amplified by PCR with the primers 9C-URA3-223 and 3CG9-URA3+880c. Second, a linker of 9Cs (5′-ccccccccc-3′) or 3CG9 (5′-cccgggccc-3′) was designed at the 3′ end of *Km*ARS primers to anneal the truncated *Km*ARSs sequences to the *ScURA3* gene prepared in the first step. Third, short truncated sequences of some *Km*ARSs were divided into two parts to design primers. One part was flanked with 9C and the other with 3CG9 in an intention to leave the central joining sequence of the *Km*ARS free after running the PCR with both primers (Fig. S1A). These steps were used to identify the minimum active sequences for *Km*ARS7, *Km*ARS11, *Km*ARS16, *Km*ARS18, *Km*ARS22, *Km*ARS36, and *Km*ARS51 by transforming the *ScURA3+Km*ARS into *K. marxianus* strain RAK3606 and selection on MM-U and replica-plating on 5-FOA. To examine whether segments of minimum *Km*ARSs can be exchanged with each other while retaining the ARS activity, a combination of primer pairs representing discrete *Km*ARSs were used to anneal them by PCR at the ends of the *ScURA3* gene as described in the third step above then followed by routine selection and replica-plating procedures (Fig. S1A).

### 2.6 Analysis of *K. marxianus* ARS consensus sequence (ACS)

To detect the ACS within *Km*ARSs, deletions and/or substitutions experiments were performed on the minimum active sequences of *Km*ARS7, *Km*ARS11, *Km*ARS18, *Km*ARS22, and *Km*ARS36. Deletion primers were designed from the minimum active sequences of *Km*ARS7 (201-250) and *Km*ARS36 (291-340) by deleting triple nucleotides at a time, while for *Km*ARS18 (111-138) primers, deletion of a single base was carried out in addition to single base substitution for all bases. In the case of *Km*ARS11 (46-105), five nucleotides were deleted at a time from the 3′ end and ten nucleotides were deleted at a time from the 5′ end. For *Km*ARS22 (991-1060), ten nucleotides were deleted at a time from either the 5′ or 3′ side.

## 3 Results

### 3.1 Autonomously replicating sequences from *K. marxianus* DMKU3-1042 (*Km*ARSs)

In this study, more than twenty-eight plasmids harboring *K. marxianus* DMKU3-1042 autonomously replicating sequences (*Km*ARSs) were isolated from the genomic libraries. Sequencing of the DNA inserts revealed that many of these plasmids with identical insert sequences, and finally, twelve plasmids were identified as having unique *Km*ARSs (Table 2). Plasmids found with identical sequence include two pRS316+*Km*ARS7, two pRS316+*Km*ARS16, two pRS316+*Km*ARS18, two pRS316+*Km*ARS36, two pRS316+*Km*ARS45, five pRS316+*Km*ARS3, two pRS316+*Km*ARS20F, and two pRS316+*Km*ARS20R. The sequences (Fig. S2) were deposited at the GenBank™ database with the accession numbers (MZ514892 through MZ514902). The size of the insert DNAs with *Km*ARSs ranged from 154 to 2,590 base pairs. These insert DNAs are distributed in the seven chromosomes of the yeast *K. marxianus* DMKU3-1042 (Lertwattanasakul et al., 2015). Four ARSs (*Km*ARS7, *Km*ARS16, *Km*ARS18, and *Km*ARS51) belong to chromosome 3, two ARSs (*Km*ARS36 and *Km*ARS20R) belong to chromosome 2, two ARSs (*Km*ARS22 and *Km*ARS20F) belong to chromosome 6, while a single ARS was identified from chromosome 1 (*Km*ARS11), chromosome 4 (*Km*ARS45), chromosome 5 (*Km*ARS3), and chromosome 7 (*Km*ARS14) (Table 2).

### 3.2 Functional validation of *Km*ARSs

**Fig. 1:**
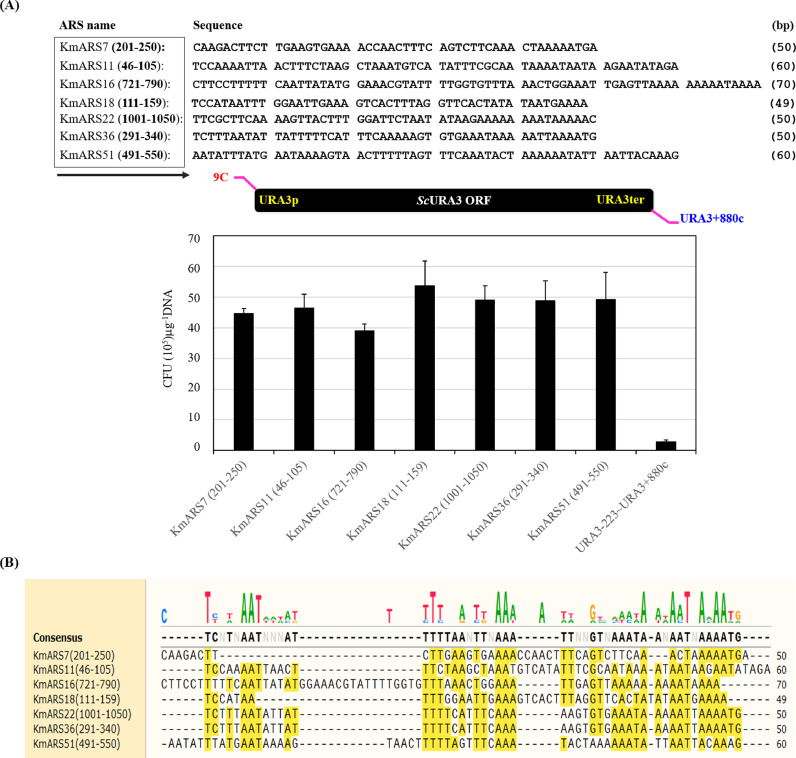
The activity of short sequences of *Km*ARSs. (**A**) Short sequences of different *Km*ARSs ranging from 49 to 70 bp are shown (top panel). Seven *Km*ARS sequences are fused to the *ScURA3* gene at its 5ˊ end and the transformation efficacies of these constructs are depicted (bottom panel). Sequence alignment and logos of the short *Km*ARSs are depicted in panel (**B**).

We have previously shown that the circular plasmid pRS316 did not replicate in *K. marxianus* DMKU3-1042 but its linear DNA efficiently integrated into the chromosomes of this strain (Abdel-Banat et al., 2010b; Nonklang et al., 2008; Hoshida et al., 2014). In this study, a simple approach based on a linear transformation protocol was adopted to concept-proof the activities of *Km*ARSs (Fig. S1A). After series of sequence alignments (Fig. S3) with known ARSs from *S. cerevisiae* (Deshpande and Newlon, 1992) and *Kluyveromyces lactis* (Iborra and Ball, 1994), *Km*ARSs sequences ranging from 21 to 70 bp were identified for replication in *K. marxianus* DMKU3-1042. To analyze the sequences more precisely, these *Km*ARSs were fused to the *ScURA3* marker gene and subjected to transformation. Upon transformation, the yeast *K. marxianus* uses its NHEJ pathway to attach the ends of these linear constructs to form circular DNA and transformants. However, some transformants may have produced by chromosomal integration of the DNA introduced. To confirm plasmid formation, transformants were inoculated on 5-FOA plates. Yeast cells with autonomously replicating DNA successfully grow on 5-FOA, while cells with chromosomally integrated *ScURA3* gene fail to grow on 5-FOA (Fig. S1A & B). Using this easy functional validation and post-transformation cellular events, truncated but functional sequences of seven *Km*ARSs were verified (Fig. 1A). The functional sequences of *Km*ARS7 (50 bp), *Km*ARS11 (60 bp), *Km*ARS16 (70 bp), *Km*ARS18 (49), *Km*ARS22 (50 bp), *Km*ARS36 (50), and *Km*ARS51 (60 bp) were shown in Fig. 1A. These *Km*ARSs replicate very effectively in *K. marxianus* giving at least 40×10^5^ CFU μg^−1^ transforming DNA. Replica plating of the transformants regularly gives more than 80% rescued colonies from 5-FOA toxicity, an indication of intracellular replication as plasmids. It is noteworthy that, the alignment of these short functional sequences showed no prominent common consensus but the AT stretches prevail the sequences (Fig. 1B).

### 3.3 Impact of truncations and triple nucleotide deletions on the activity of the region 201-250 of *Km*ARS7

**Fig. 2:**
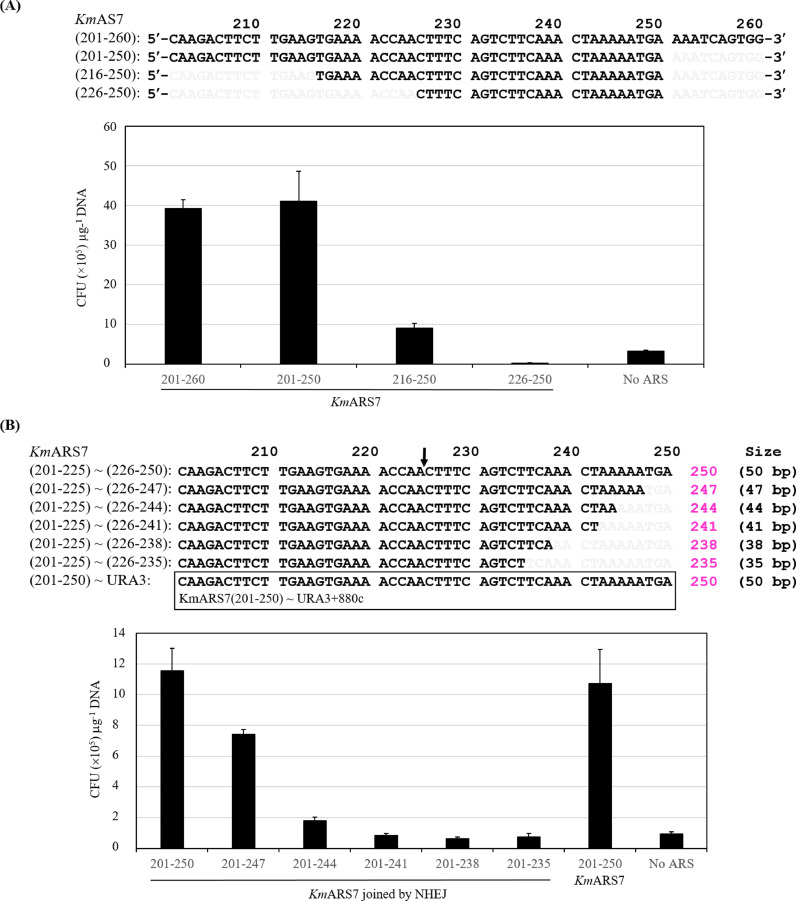
Impact of truncations and nucleotides deletions on *Km*ARS7. (**A**) *Km*ARS7 (201-250) and its truncated fragments were fused at the 5ˊ end of the marker gene (top panel). A chart for the transformation efficacy of the regions *Km*ARS7 (201-260) and *Km*ARS7 (201-250) and its truncated fragments is depicted (bottom panel). (**B**) Sequences of *Km*ARS7 (201-205) and its triple nucleotides deletion fragments are shown on the top and their corresponding transformation efficacies are depicted on the bottom. The values of transformation efficiencies (CFU μg^−1^ DNA) in (**A**) and (**B**) are due to the use of different preparations of RAK3605 competent cells. Therefore, the charts in (**A**) and (**B**) represent the general patterns of transformability of *Km*ARS7 (201-250) and its truncation and deletion products.

We have previously demonstrated that 60 nucleotides of *Km*ARS7 (201-260) effectively drove the replication of the *ScURA3* gene (Hoshida et al., 2014). However, the *Km*ARS7 retains its potent activity even after further truncations of this region. The region 201-250 gave an average of 40×10^5^ colony-forming units (CFU) μg^−1^ transforming DNA, but the number of transformants was dropped drastically when the regions 216-250 or 226-250 were transformed in conjunction with the *ScURA3* gene (Fig. 2A). Truncations of the region *Km*ARS7 (201-250) were also investigated by triple nucleotide deletions. Two separate primers for each construct were used to amplify the *ScURA3* marker. One primer was *Km*ARS7 (201-225)c-3CG9 and the other set of primers were *Km*ARS7 (226-250)9C and its triple nucleotide truncations (Fig. 2B). Deletion of three nucleotides from the 3′ end of *Km*ARS7 (201-250) resulted in the reduction of the transformation efficiency on average by about 25%, while further deletions reduced the transformability of *Km*ARS7 to a level comparable to the transformation of *ScURA3* gene only. Colonies that appeared on the plates transformed with *Km*ARS7 (201-244), (201-241), (201-238), and (201-235) are mostly the result of integration activity rather than autonomous replication as judged by replica plating on 5-FOA (data not shown).

### 3.4 Functional characteristics of *Km*ARS11

**Fig. 3:**
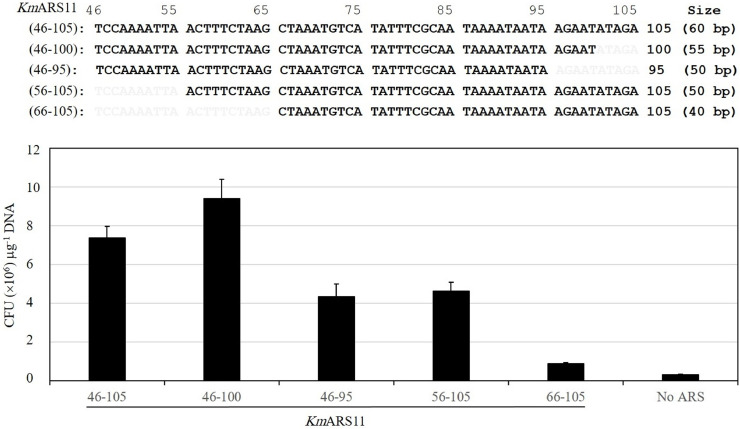
Effect of 5′ and 3′ deletions on the activity of the region (46-105) of *Km*ARS11. Nucleotide sequences of *Km*ARS11 (46-105) and its deletion fragments are depicted on the top. A chart of transformation efficiencies of ARSs into the RAK3605 competent cells is shown on the bottom.

The whole insert sequence of *Km*ARS11 is 154 bp. The regions *Km*ARS11 (46-105) and (46-100) gave comparable high transformability, while the transformability of the regions *Km*ARS11 (46-95) and (56-105) was declined but produced significant levels of transformants compared with transformation without any ARS. The transformability of the regions *Km*ARS11 (66-105) was not distinct from that of the *ScURA3* gene (Fig. 3). As a result, 50 bp of *Km*ARS11 (56-105) retains the transformability.

### 3.5 Functional characteristics of *Km*ARS18

**Fig. 4:**
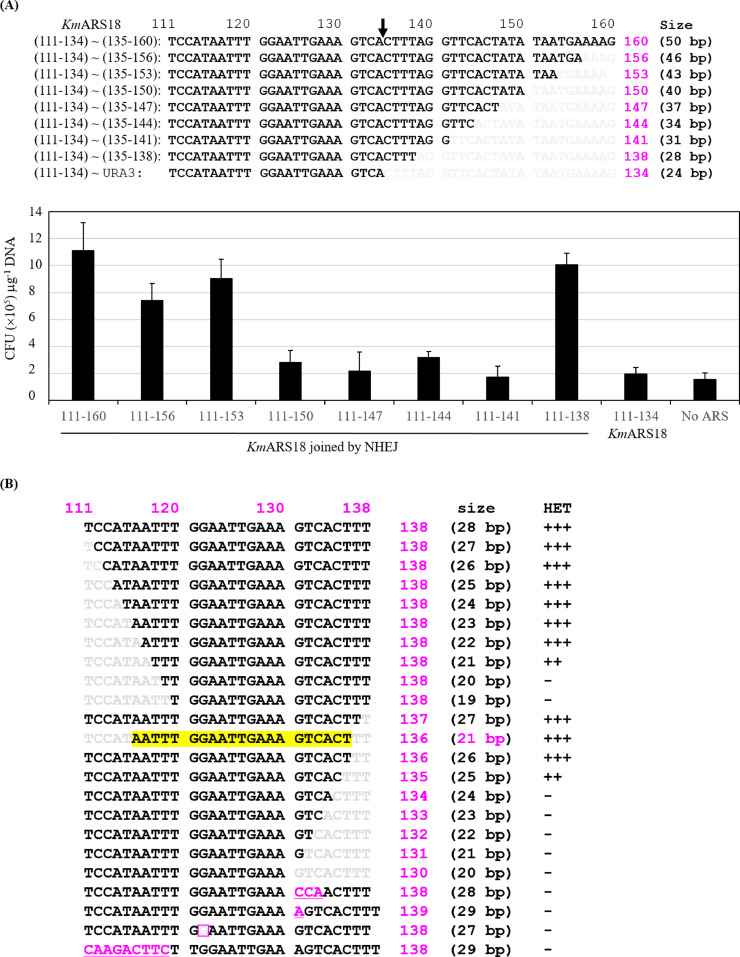
Impact of nucleotides deletion on the activity of the regions *Km*ARS18 (111-160) and *Km*ARS18 (111-138). (**A**) Sequences of *Km*ARS18 (111-160) and its successive deletion fragments are shown on the top. The downward arrow indicates the position that separates the two primers for each construct. The transformation efficacy of *Km*ARS18 (111-160) and its deletion fragments are shown on the bottom. **(B)** Impact of single nucleotide deletion on the activity of *Km*ARS18 (111-138). The sequences that produce highly efficient transformation (HET) are indicated by (+++), active regions after the deletion are indicated by (++), and the regions lost the activity are indicated by (-). The minimum sequence of *Km*ARS18 with highly efficient transformability is indicated in yellow background. Other modifications presented are the replacement of GTC with CCA, the addition of A at position 131 of *Km*ARS18 (111-138), deletion of G at position 122 of *Km*ARS18 (111-138), and replacement of the nucleotides from 111-119 of *Km*ARS18 (111-138) with sequences from *Km*ARS7. These modifications are shown in sequences # 20, 21, 22, and 23, respectively.

**Fig. 5:**
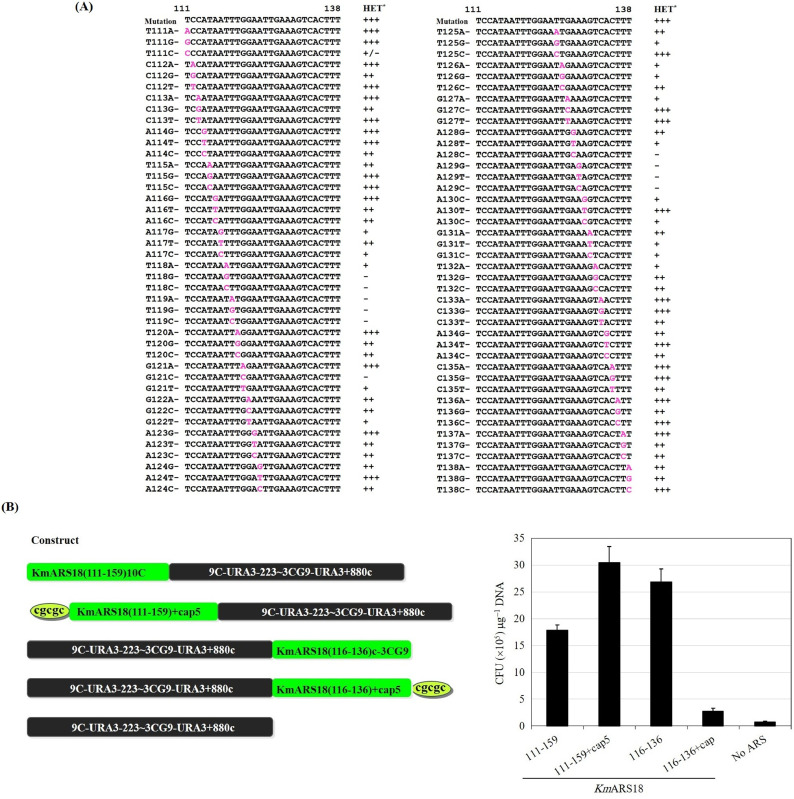
Effect of nucleotide substitutions and addition of cap sequences on the function of *Km*ARS18 regions. (**A**) Influence of single nucleotide substitutions on the transformability of *Km*ARS18 (111-138). Sequences with the symbol (+++) give a highly efficient transformation, those with the symbol (++) give moderate transformation, those with the symbol (+) give weak transformation, and those with the symbol (-) completely lost the activity. The sequence with (+/-) give variant transformability (mainly small colonies). **(B)** Sensitivity of *Km*ARS18 (116-136) to cap. The addition of cap sequences at the end of *Km*ARS18 (116-136) adversely affects the ARS function of this region. The addition of cap “cgcgc” to the region *Km*ARS18 (111-159) positively enhanced the transformability, while the transformability of the capped *Km*ARS18 (116-136) is greatly declined relative to the uncapped same region.

Deletion of seven nucleotides from the 3′ end of *Km*ARS18 (111-160) slightly decreased the transformation efficiency of *Km*ARS18. Further triple-nucleotide deletions resulted in the reduction of the transformation efficiency of *Km*ARS18 on average to levels as low as 34%. Surprisingly, the region *Km*ARS18 (111-138), which is 28 bp-long, showed elevated transformation efficiency (Fig. 4A). This encouraged us to look for extra ARS active sequences from *Km*ARS18. The regions *Km*ARS18 (111-138) [28 bp], *Km*ARS18 (139-159) [21 bp], and *Km*ARS18 (121-149) [29 bp] were tested for transformability. Both the region *Km*ARS18 (121-149) and the longer *Km*ARS18 (139-159) showed reduced transformation efficiency relative to *Km*ARS18 (111-138) (data not shown). This region, *Km*ARS18 (111-138), was thoroughly investigated by single nucleotide deletion from both sides (Fig. 4B). The deletion of seven nucleotides from the 5ˊ end (TCCATAA) resulted in the generation of fewer transformants. Moreover, an additional single nucleotide deletion from this region completely abolished its transformability. On the other hand, the deletion of four nucleotides from the 3ˊ end (135-CTTT-138) resulted in the elimination of transformability. The region as short as 21 bp-long of *Km*ARS18 that covers the nucleotides (116-136) was capable to drive efficient transformation (Fig. 4B). Replacement of three nucleotides 131-GTC-133 with CCA, the addition of A at the position 131, deletion of G at position 122, and replacement of the region *Km*ARS18 (111-TCCATAATT-119) by the introduction of nine nucleotides of *Km*ARS7 (201-CAAGACTTC-209) at the same site negatively affect the transformation efficiency of the region *Km*ARS18 (111-138) (Fig. 4B). Furthermore, as shown in Fig. 5A, a single nucleotide substitution in the region *Km*ARS18 (111-138) induces moderate to weak effect or complete loss of transformability. However, the substitutions at some sites did not affect the transformability and the mutants gave transformants similar to the original sequence. Substitution at the sites T118G, T118C, T119A, T119C, G121C, A128C, A129G, A129T, or A129C made the *Km*ARS18 (111-138) lose the ability to develop transformants (Fig. 5A). In other cases, very few but small transformants were developed upon base substitution at the sites T111C, A117G, A117C, T118A, G121T, G122T, T125G, T126A, T126G, G127A, A128T, A130C, G131T, G131C, or T132A (Fig. 5A). Additionally, the region of the 21 nucleotides [*Km*ARS18 (116-136)] that showed highly efficient transformation (Fig. 4B) was capped by adding five nucleotides, “CGCGC”, at its free end after joining it to the marker gene. Transformation of this construct and a similarly capped region *Km*ARS18 (111-159) as a control, revealed that the region *Km*ARS18 (116-136) is very sensitive to additional bases at its 3′-end (Fig. 5B) but 5′-capping by the “CGCGC” did not interfere with the efficient transformability of the region *Km*ARS18 (111-159) [49 bp].

### 3.6 Functional characteristics of *Km*ARS22 and *Km*ARS36

**Fig. 6:**
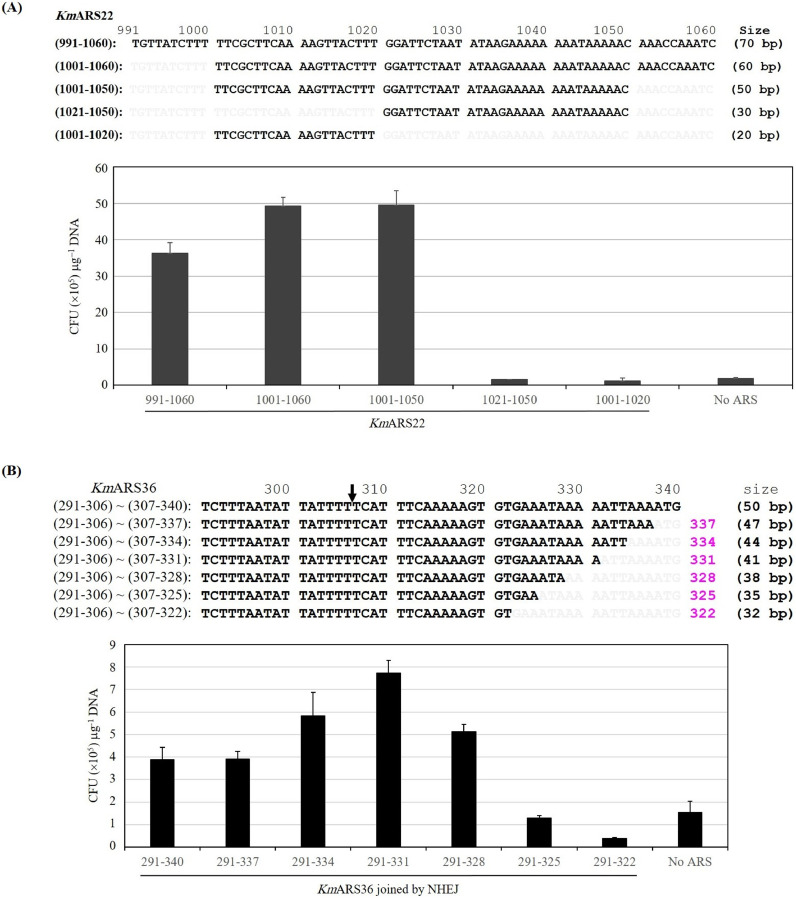
Impact of truncations and nucleotides deletions on *Km*ARS22 and *Km*ARS36. (**A**) Effect of 5ˊ and 3ˊ ends truncations on *Km*ARS22. Sequences of *Km*ARS22 (991-1060) and its truncated fragments are shown on the top. These sequences are attached to the *ScURA3* marker gene and the transformation efficiencies are shown on the bottom. **(B)** Influence of nucleotides deletions on *Km*ARS36. The region *Km*ARS36 (291-340) was divided into two primers as indicated by the downward arrow. Each primer was attached to one end of the *ScURA3* marker gene. Sequences after successive deletions are shown (top panel). A chart for the transformation efficiency of the *Km*ARS36 (291-340) and its deletion variants is shown (bottom panel).

The region of *Km*ARS22 (991-1060) [70 bp] with high replication propensity was truncated (Fig. 6A). Regions of *Km*ARS22 (1001-1060) [60 bp] and *Km*ARS22 (1001-1050) [50 bp] behave similarly as effective replicators, while the regions *Km*ARS22 (1021-1050) [30 bp] and *Km*ARS22 (1001-1020) [20 bp] separately failed to drive the replication process (Fig. 6A). The region (1001-1020) [20 bp] complements the region (1021-1050) [30 bp] to retain the function of *Km*ARS22 (Fig. 6A).

The region (291-340) of *Km*ARS36 [50 bp] gave an average of 3.9×10^5^ CFU µg^−1^ DNA. Contrary to the other *Km*ARSs, the transformability was increased gradually upon deletion of triple nucleotides at a time and reached up to 7.73×10^5^ CFU µg^−1^ DNA when nine nucleotides were deleted from the 3ˊ end leaving a region of 41 bp [*Km*ARS36 (291-331)]. When twelve nucleotides were deleted leaving a region of 38 bp [*Km*ARS36 (291-328)], the transformability was slightly declined compared with *Km*ARS36 (291-331) but showed higher transformability than *Km*ARS36 (291-340), indicating that the 38 bp-long region is still capable to drive the autonomous replication. Further deletions from the 3ˊ end, leaving the regions 291-328 or 291-325, caused the loss of transformability (Fig. 6B). The nucleotides that covering the region 326-ATAAAA-331 are indispensable for the activity of *Km*ARS36.

### 3.7 Impact of *Km*ARSs interchanged sequences on transformability

There is clear variation in the sequences among the identified core sequences of *Km*ARSs (Fig. 1B) and these *Km*ARSs have no sequence identity with the optimized *Kl*ARS (Liachko and Dunham, 2014) (Fig. S4). Although sequences of the regions *Km*ARS18 (1181-1240) and *Km*ARS7 (123-182) have sites with fairly high identity to other yeast ARS consensus sequences (ACS), these regions did not drive efficient transformability relative to their corresponding regions of the *Km*ARS18 (111-159) and the *Km*ARS7 (201-250) (Fig. S5). Due to the disparities in the consensus and lengths of the identified *Km*ARSs, short sequences of these ARSs were interchanged with each other to judge whether or not they could induce efficient transformability. As shown in Table 4, the majority of various regions of the *Km*ARSs when interchanged, they generate in some instances even more transformants than do the corresponding regions of individual *Km*ARSs. The most prominent results were the highly efficient transformability of *Km*ARA18 (111-138) when interchanged with six other *Km*ARSs namely *Km*ARS7 (230-250), *Km*ARS11 (61-100), *Km*ARS16 (753-790), *Km*ARS22 (1021-1050), *Km*ARS36 (307-340), and *Km*ARS51 (516-550). Interchanged regions of *Km*ARS11 (61-100), *Km*ARS16 (753-790), and *Km*ARS36 (516-550) respectively with *Km*ARS7 (201-229), *Km*ARS11 (46-60), *Km*ARS16 (721-752), *Km*ARS18 (111-138), *Km*ARS22 (1001-1020), *Km*ARS36 (291-306), and *Km*ARS51 (491-515) also induced highly effective transformability (Table 4). Meanwhile, these interchanged sequences showed fewer consensus identities and the similarities mainly skewed towards the 3′ and 5′ ends without clear consensus in the middle (Fig. S6). It is noticeable that transformants from the interchanged constructs gave between 81 to 100% colony growth on 5-FOA.

**Table 4:** Influence of interchanged sequences of *Km*ARSs on the ARS activity.

ARS fused at the 3ˊ end of *ScURA3*	ARS fused at the 5ˊ end of *ScURA3*						
	*Km*ARS7 (230-250)	*Km*ARS11 (61-100)	*Km*ARS16 (753-790)	*Km*ARS18 (139-159)	*Km*ARS22 (1021-1050)	*Km*ARS36 (307-340)	*Km*ARS51 (516-550)
*Km*ARS7 (201-229)	49*	44.2	53.2	1.55	41.45	32.5	22
*Km*ARS11 (46-60)	41.45	44.35	60.25	2.7	3.0	55.1	26.95
*Km*ARS16 (721-752)	3.0	63.85	41.55	2.0	1.7	10.9	1.85
*Km*ARS18 (111-138)**	67.85	57.4	63.05	45.8	61.95	61.1	66.4
*Km*ARS22 (1001-1020)	4.05	38.75	41.65	1.95	28.65	30.8	45.4
*Km*ARS36 (291-306)	1.9	55.1	59.15	2.0	2.7	11.85	2.3
*Km*ARS51 (491-515)	8.2	51.9	40.35	36.75	38.75	38.45	51.3

*Transformation efficiencies of the interchanged ARS sequences are tabulated as CFU (×10^5^) μg^−1^ DNA. Using the same lot of yeast competent cells (RAK3605), the marker gene alone gave approximately 1.26×10^5^ CFU μg^−1^ DNA. **Tested colonies from transformants of the *Km*ARS18 (111-138) in combination with all other regions of *Km*ARSs that shown in this table gave 81 to 100 percent growth on 5-FOA.

## 4 Discussion

Autonomously replicating sequences (ARS) are the replicator elements to which bind the initiator protein that unwind the DNA double helix and recruits additional factors to initiate the process of DNA replication. The proteins that regulate replication are highly conserved, including the origin recognition complex (ORC), which binds directly to replication origin sequences, but Gilbert (2001) stated, “In several eukaryotic replication systems, it appears that any DNA sequence can function as a replicator”. However, many studies on yeast ARS helped to define specific sequences that function as origin replicators in *S. cerevisiae, S. pombe, K. lactis*, and *C. guilliermondii* (Stinchcomb et al., 1980; Clyne and Kelly, 1995; Irene et al., 2004; Liachko et al., 2010; Liachko and Dunham, 2014; Foureau et al., 2013). Here we report the identification of twelve functional *Km*ARS from the strain DMKU3-1042 capable to replicate plasmid DNA but have no common consensus sequences.

Previously, Iborra and Ball (1994) reported the isolation of three small DNA fragments from *K. marxianus* strain ATCC12424 [ARS1 (1267 bp), ARS2 (1206 bp), and ARS3 (1200 bp)]. ARS1 and ARS2 contain both ARS and centromeric elements, while ARS3 contains ARS core sequence only and all function in *K. lactis*. Only two of our *Km*ARSs identified in the current study share identity with ARS1 and ARS2 from the strain ATCC12424. One of them is *Km*ARS3 (Table 2), which shares 89.53% identity to ARS1 and the other is a portion consists of 128 nucleotides from *Km*ARS20F that shares 100% identity to ARS2. However, none of the *Km*ARSs reported here share significant identity to the ARS3 from the strain ATCC12424, which indicates that in this study ten *Km*ARSs are identified for the first time from *K. marxianus*. This might be either the ARS3 replicator is not functional in *K. marxianus* DMKU3-1042 or its rival was missed during our libraries' screening. It has been reported that very similar ACS of nonanucleotide (5′-TTTATTGTT-3′) is common between *K. marxianus* and *K. lactis* (Iborra and Ball, 1994). However, this same ACS is not found in any of the currently investigated *Km*ARSs.

In this study, we also identified minimal sequences that function as ARS. These sequences indicated again that ACS found in *S. cerevisiae* and *K. lactis* does not exist in the *K. marxianus* ARSs. In addition, generally within 50-bp *Km*ARS sequences, at least 21-bp are functioning as ARS for plasmid replication. Among the identified minimal functional sequences, any clear consensus sequence was not found, indicating that the essential sequence of ARSs in *K. marxianus* are divergent.

With some exceptions, the majority of the interchanged sequences for *Km*ARSs replicate effectively. The sequences 5′-AAA(G/A)T(×××)(T/A)TT-3′ and 5′-AAAA(T/A)AAAAAT-3′ are likely the common consensus in the interchanged *Km*ARSs of the strain DMKU3-1042 (Fig. S6) and their sequences position weight matrix logo (Crooks et al., 2004; Liachko et al., 2010) suggest the prevalence of poly-A at the 3ˊ termini of the interchanged sequences. It is noteworthy that, the transformability of the regions *Km*ARS7 (123-182) and *Km*ARS18 (1181-1240) is completely different from its counterpart regions *Km*ARS7 (201-250) and *Km*ARS18 (111-159) (Fig. S5). Because the regions *Km*ARS7 (123-182) and *Km*ARS18 (1181-1240) did not function as effective replicators though they contain sites that share remarkably high similarity to the ACS of *K. marxianus* strain ATCC12424 (Iborra and Ball, 1994) and ACS of *K. lactis* (Liachko et al., 2010).

## 5 Conclusion

Identification of the short sequences that function as *K. marxianus* autonomous replication origins using a novel and simple approach for the validation of the ARS function. ACSs of *K. marxianus* DMKU3-1042 are diverse among the *Km*ARSs as well as from those of *K. lactis*, indicating that eukaryotic replication systems are not necessarily having common ACS. That is evidenced by the fact that no site-specificity was detected in early embryos of frogs, flies, and fish (DePamphilis, 2003). However, mammals contain genetically required sequences that convey origin activity when translocated to other chromosomal sites, but they lack identifiable, genetically required consensus sequences such as ACS in budding yeast replicators (Prioleau et al., 2003). A single nucleotide mutagenesis approach helps to identify specifically the essential nucleotides within the span of the active *Km*ARSs. The *Km*ARS18 ACS termini are very sensitive to nucleotides substitution. All defined minimum active *Km*ARSs, except *Km*ARS22 and *Km*ARS16, are located at the intergenic sequences of the genome. Overall, the minimum *Km*ARSs reported here are capable to induce the formation of circular DNA and effectively replicate within the yeast cells. The *Km*ARSs described in this study will provide additional options that are versatile and more effective to develop large sets of molecular tools for better engineering of this strain.

## Authors’ contributions

**BMAA and RA:** Conceptualization. **BMAA:** Methodology, Investigation, and Validation. **BMAA and HH:** Writing- Original draft preparation. **BMAA, HH, and RA:** Writing- Reviewing and Editing. **HH and RA:** Resources. **RA:** Acquisition of the financial support for the project leading to this publication.

## Ethical approval

This article does not contain any studies with human participants or animals performed by any of the authors.

## Declaration of Competing Interest

None

## Appendix Supplementary materials

Image, application 1
