# Supplementary material for: Various short autonomously replicating sequences from the yeast Kluyveromyces marxianus seemingly without canonical consensus
Source: Curr Res Microb Sci. 2021 Jul 31;2:100053. doi: 10.1016/j.crmicr.2021.100053 (PMC8610295; doi:10.1016/j.crmicr.2021.100053)
Supplement: Supplementary file 1 [file mmc1.docx]

**Supplementary Material**

**Various short autonomously replicating sequences from the yeast *Kluyveromyces marxianus* seemingly without canonical consensus**

Babiker M.A. Abdel-Banat^a*,b^, Hisashi Hoshida^c*^, Rinji Akada^c^

^a^Date Palm Research Center of Excellence, King Faisal University, Al-Ahsa 31982, Saudi Arabia.

^b^Department of Crop Protection, University of Khartoum, Shambat 13314, Sudan.

^c^Department of Applied Molecular Bioscience, Yamaguchi University Graduate School of Medicine, Tokiwadai, Ube, Japan.

*Corresponding authors:

Babiker M.A. Abdel-Banat

Date Palm Research Center of Excellence, King Faisal University, Al-Ahsa 31982, Saudi Arabia. Email: [babikera@hotmail.com](mailto:babikera@hotmail.com); [bahmed@kfu.edu.sa](mailto:bahmed@kfu.edu.sa)

Hisashi Hoshida

Department of Applied Molecular Bioscience, Yamaguchi University Graduate School of Medicine, Tokiwadai, Ube, Japan. Email: [hoshida@yamaguchi-u.ac.jp](mailto:hoshida@yamaguchi-u.ac.jp)

**
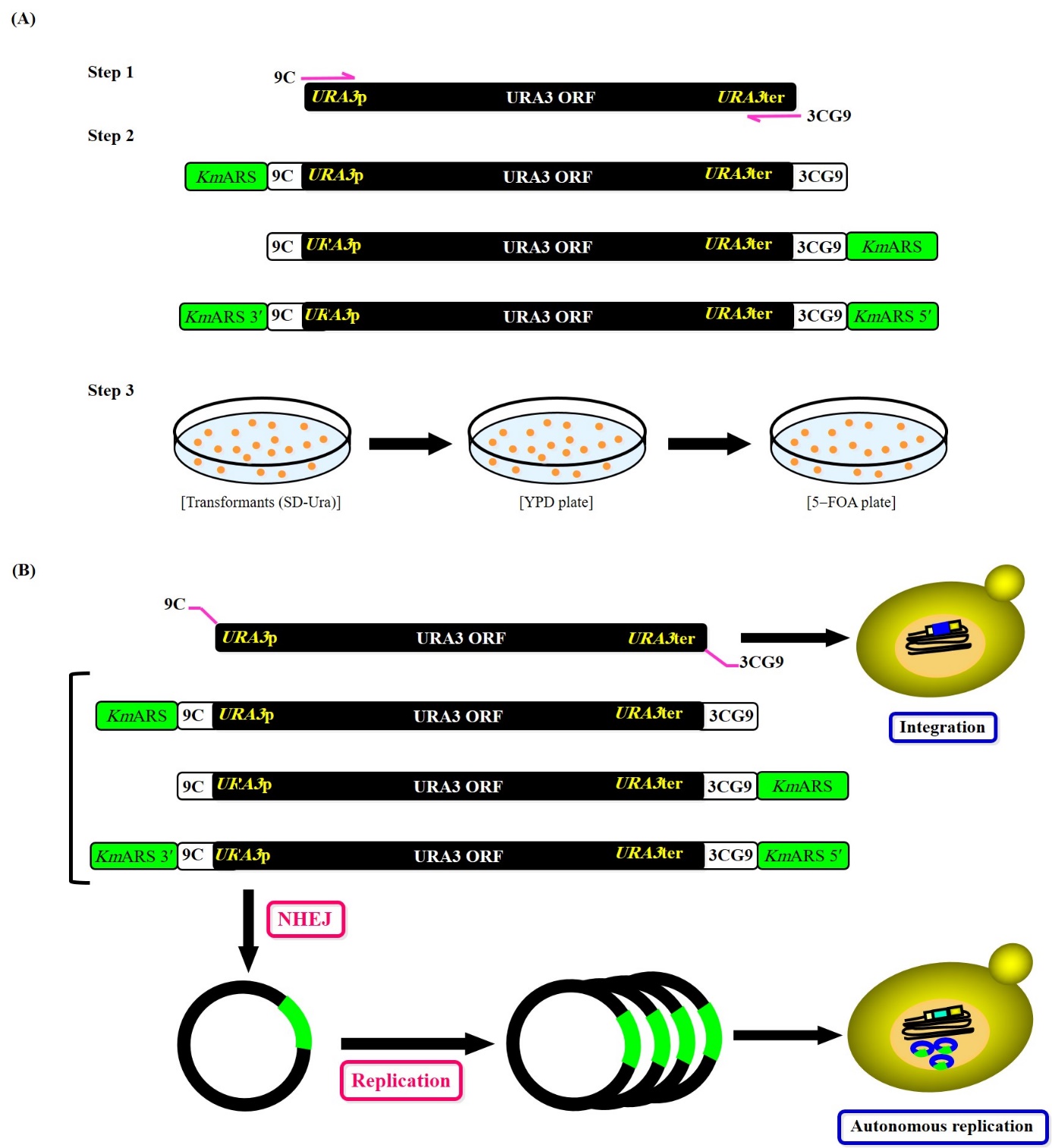
**

**Fig. S1.** ARS functional validation approach and cellular events post-transformation. (**A**) Schematic representation of fusion PCR steps to amplify the URA3 marker gene with *Km*ARSs attachments at its ends and the constructs transformation into *K. marxianus* DMKU3-1042 and selection. (**B**) Schematic representation of likely the pathways the linear DNA undergoes inside the cells of *K. marxianus* DMKU3-1042 after transformation.

***Kluyveromyces marxianus* DMKU3-1042 DNA, autonomously replicating sequence, *Km*ARS7**

Note="*Km*ARS7 (201-247); minimum sequence for autonomous replication"

AGAATTCAGG GATGATCTTG AGAAGTTCTT AGAGTCTTAC GAGGGAACAG AAGATTTGGA 60

ACCAGCCAAA GCTGCTATAG CGGAAGCAGA TATTTTGCTA AGTAAATGAT TAAATAATTA 120

AATATGTGGA AATACATTAA TCTTTTTATA TTTTTGCAGT TCGTTGTCGC TATAATTTAT 180

AGTCATCTCG TTAGTTCAAA CAAGACTTCT TGAAGTGAAA ACCAACTTTC AGTCTTCAAA 240

CTAAAAATGA AAATCAGTGG AAGAAGGTAA ACGACTTCAT GTTATATATG AATTGAATAG 300

TAATGGAAAT AACCAAAAAC AGCTCAACAG AAAACAAACA AAATACGTTA AGACCTGAAC 360

TCCTAGCAGA ACCATAACTG CCAAATATTT ATTATCTGTG GAGATCTTAT ATTCTAAAAC 420

CAAAAAAAAT ATAACTTAAA AGTTAAAAAG AAGATGTTCT AACTGAGGTT CGAACTCAGG 480

ACCTTTGCCG TGTGAAGGCA ACGTGATAGC CACTACACTA TTAGAACTAC CTTATGGGAA 540

AAAGAAAAAT AGAGTACAAC TAGAATGGTA AGATCTGTGA CCTTTTCTAA ACACTTAATT 600

CCATATAGAC AGTTCCCACC CACCATAAGG TCACAATTAT AATGTCTTTA GAAGACCACT 660

GTCGTTCATC ATCTTCCTAA GCCCTCTCTC TAAAGCGGCA TATTTCCGTA ATTTGTTCTT 720

CTTTGCACAG GCACGTGAGA TGACTCCGAT TATTCCCACA TGCATATTTA GCCTCTCTAG 780

GGGCTCGAG 789

***Kluyveromyces marxianus* DMKU3-1042 DNA, autonomously replicating sequence, *Km*ARS11**

Note="*Km*ARS11 (46-95); minimum sequence for autonomous replication"

GAATTCCCAA AATCAATGAT TCATACACTT TTTACACTGT GACGTTCCAA AATTAACTTT 60

CTAAGCTAAA TGTCATATTT CGCAATAAAA TAATAAGAAT ATAGATATCA AAGGTCTGTG 120

AAGCTTTTAT TTACACTAAT AGATATTCTC GAGG 154

***Kluyveromyces marxianus* DMKU3-1042 DNA, autonomously replicating sequence, KmARS16**

Note="*Km*ARS16 (721-770); minimum sequence for autonomous replication"

GAATTCAGGC AAAGAGAGCT GTTGCACAGA AGCTTTTTAG GAAACTTAAA TTGTCAAGCA 60

GACACAAATG TTACATTTTT TGTGTTGGAT GCAACAACAT GGTTGAGACA TTTTGCGCAT 120

ATCTATAAGC TTGCCACCAG TAATGTTTTG AAATTTGCCA TTTGTTTAAC AACTTTCCAG 180

GAGTTGAGAT TTCTTCGTAA ATCGAAGGAT GAAAGCGTAT TAGAGGCCGC CACTAGAGCA 240

GTCATAGCAG TGAGACAATT ATACTATGAA CGAAACCTTC TTGCTTTGAG ATTTACGGGT 300

AACGTTGCTG GACACTTGGA GGAACACCTA GAGATCGAAG AACAAATGAC ATGGAAATCA 360

CATGTGGATG AGTTTGTCAT AGATGCAATT GCAAAAGCAC AAGAAAAGTT CAACGTTTTA 420

AACAACGATG CAATGCAGAA CGGAAAAGAC TGTATCCCTG TATCTAGCGA TACCCAAGAT 480

CCCAAGAAGT TTAATTTCAT CAGTTTAGTT ACTGACGACT TCAATATGAG AAATAAAGCT 540

CAGCAATTGG GGATTCGTAC TTTCAGTACT AGATTTGTAT TCGCCGTGTG CAGGGAACTT 600

GGTAGAGAAG CAGGTGTATG TACTAATTAA ATCTATTCAT ATATCATATC TTAACGAACA 660

GACGTTTGTT TATTTTATAC ATTTTTAAAC CTCTAATTTG ACTAATTAAT TCTACAACTA 720

TTTTATTTTT TTTTAACTCA ATTTCCAGTT TAAACACCAA AATACGTTTC CATATAATTG 780

AAAAAGGAAG TCATGGTTAC ATTGAACGTA ACTGGCGTTG GAGGAGCAAA GACCTTGCAA 840

GATAATTGTA CTAAATTTTG TATGATCCGT TTTCAATAGC GTTAGTTTAT CATTTAAATT 900

ACAATTTTGC TTTATCTTGA CACGCAACCA ACATTTATCT GAACTAGTCT CTCTCATAAT 960

TCGTATTTTG GTTGAGGATA GACAAAGGCT GGACTTGACC GCGGCGGAAG AGTTCTGACT 1020

TGATGCATCA AAACCCATTA ACAAATTGGT AGCCATTGGA GCTGTGTGGG TTTTTTTCCT 1080

GGTAATACAT CTAGCTTTGC TTGCAACCTT CGACTTTTCT ATTAGTGAAT TTATCGATCC 1140

TTTGCCATTG CCGCCATCAC GAGAACTTTT ATTATAGGAT TCTCGAG 1187

***Kluyveromyces marxianus* DMKU3-1042 DNA, autonomously replicating sequence, *Km*ARS18**

Note="*Km*ARS18 (116-136); minimum sequence for autonomous replication"

AATTCAATAA GGTTTTTTTT GGTTTTATGG AATGGAATTT GAGTGTACAT GTGTTGTTAA 60

TGGAATGTTG TAAACAAGTG ATTATTATAA GGCATAATGC CAGGAATCTT TCCATAATTT 120

GGAATTGAAA GTCACTTTAG GTTCACTATA TAATGAAAAG AACACGATAA TAAAAACTCC 180

GGGATCCGCT AATATATTTA ATGATACACA GGCATACATA CATACATACA TACAAACAAA 240

AACACACATA TAAATATTTC TCGTTAAGTG TTCAGATAGG AGGTTCACTT TCTAGCTCCT 300

TTAATCTCAT CTGTTCTAAT TCTTGTTTGT CATCGGCAAT AGTAGGCAGA GCAAAGTTGT 360

TAAAGTTGCT GCTGTAAGAG GGTACTGGAT CCGTCGGGTA GCTCATAGCG ATATCACTTT 420

CCGAAAGTGT AACTTCTGGC AACGTAGACA TGTTCTGTTG TAGGAGGTAT CCATAAACCT 480

GGTCAATGGG TGAACTTGGT TCTTCCAATG AAGTATTTGC CGCATTGTCG TTCGTGGTTA 540

CATGTCCATT ATCCTGGGCG ACCGGAACAC CTTCTGCTAC AGGTGACTCC TGCTGCTGCT 600

GCTGTTGCTG TGGGATTTCT CTATGCGTCC CAAGCACGAT TTCGATAGAC ATACCAGTGA 660

CATTTCTGAG ACGTTTCAAA CGATCAACGT TAATCAAATC TTGAAAGAAA GTGACGGACT 720

CATCTTCTCC AGCGGAGTTG TGATCTGAAG CAATCAGTTT TGGAATGAAC GAGAGTTTTT 780

CCGACTGTGT TGGGATATCG CTTGTGCGTT GACCACCCAC CAATCTGTTT GATTCTGTAT 840

AAACCTGATT CTTCTTGGAC AAGTTTGCCA AGACTTCAAC GTAATATTGG AAAGTGAAAA 900

ACTTGTTTTT GACGTCCGAG GTTGGGAAAG TGTCTAAGGG GACCTTTAGC TTTAGCATTA 960

CCGATGCATC ATGGGTATCT GGGTCTGTAT ATAGTGGTGC CACGCTTTGG CATATGTCTT 1020

TTCTAAAAGT CTCTGTTTGC TCTAGGTTTG CAGAATTAGC AACTCTGCAA ATCCTTACCA 1080

AAGTAGCGAT CAAGCCTGCT GGATGCGAAT AATGTTTATA ATGCGAGACT TTGACTTTGA 1140

CTTGGATATC TTCCCCTACA GTGTATCCGA GAGACGGGAT TTCTACGGAA ATCTTGACTG 1200

TTTTGTTGTT GTTGTTGGTG TTATTATTAT TATTATTATT GTTGTTGTTA CTGGTGCTCG 1260

CGCTGCTATT GTTATTGTTA TTGTTGGTAC TATTATTATT ATTATTGCTA TTATTTTCTG 1320

CGGGACAAGA GTTTCCTGAG TTACTCGAG 1349

***Kluyveromyces marxianus* DMKU3-1042 DNA, autonomously replicating sequence, *Km*ARS22**

Note="*Km*ARS22 (1001-1050); minimum sequence for autonomous replication"

GAATTCAAAT GGTATATGTT TAATGACTTC TTGGTTGAAG AGATTGAGGA AGAAGAAGCC 60

CTCCGTATCT CATACTGGTG GAAGACACCT GAAATTGTCG TTTATTCTGA TGCAGAAGAG 120

ATTAGAAAGC CTTTTGTTCC AGTTTCAGAA TACAGTATAG ACGATAATAT ATTATATCGT 180

GATTACTTCA GTGAAGGTAT AAGGAAAGAT GTCGTTAGGC AATACACATT ACTTACTAGA 240

GAGGAGCCAC CTGGACCTGG GACCTTAGTG GCCCTCGATG CAGAGTTTGT GTCCTTGACT 300

GAACCTCGAT TAGAAATTAA TTGTAAAGGT ATGAAGACTC TATTAAAACC TGCAAAGAAA 360

TCTTTAGCAC GTGTGTCACT TTTACGTGGA GAGGGAGAAC TTGAAGGTGT ACCTTTTATC 420

GATGATTATA TTATAAGCGA GTGTCATATT GAAGATTATC TAACTCAGTT CAGTGGAATT 480

GAACCGGGTG ATTTAGATCC TAAGTTGAGT AAAAAGAGCT TGGTAAAGAG ACAAGTCTTT 540

TATAGAAAGA TCTGGCTACT CCTTCAATTA GGCTGTGTTT TTGTTGGGCA TGGTTTAACC 600

AATGATTTCC GCCAGATTAA TATTCATGTT CCTGCTTCTC AAATCAGAGA CACATCTCTC 660

TATTATTTGA AGGGTAAGAG GTACTTGTCG TTACGTTATT TGGCATATGC AGTATTGCAT 720

AAACAAGTCC AAACTGGGAA CCACGATTCC ATTGAAGATG CACACACAGC CTTACTGTTG 780

TACAGAAAAT ACTTGCAACT GAAGGAAGAA GGTGTCTTCG AAATGTACCT TGAAAATATT 840

TATGATGAAG GCCGGAAAAT GGGATTTAGA GTTCCTGAAC AGTATCCTAT GTAGAATTAT 900

ATATCATCGA TTATAATACG TTTAGTAGTA GAGGTTTGAA ACCGTTTAAT TGATTCGTTA 960

AAAACAACAT GTTGTTCTAG TTTTAGATAG TGTTATCTTT TTCGCTTCAA AAGTTACTTT 1020

GGATTCTAAT ATAAGAAAAA AAATAAAAAC AAACCAAATC AAATGAGAGT TGATGAAATA 1080

AACAAAGATA TAATACGCTT ACCACTACCG AATATAAATA TATCAAGAAT GCTGCTGACC 1140

CAACACATAC TCCATTCTTC TATTAACAGC TCGAG 1175

***Kluyveromyces marxianus* DMKU3-1042 DNA, autonomously replicating sequence, *Km*ARS36**

Note="*Km*ARS36 (291-328); minimum sequence for autonomous replication"

AATTCAATCT ACTCACGAGG TTAGTGCTGG TTGTGTCAAT TTGATTAAAA AAATACTTAA 60

CAGATCAGTG GCCAAAAGAC CAACTATAAA TGAAATAGTG GAAGATCCAT GGCTCGCATT 120

ATAAAATTCG ACCAATTAAA TTTTTTTAAA ATAAAAACGC TATAATACGT GAGTATTGTG 180

CATGTCGATA TACATCTGCT TTTTTGACAA TCCTAAGAAT TATAATCCGC CTTAATAATT 240

TAATTACCTC ATATATAATA TATAATTGCC ATATATTTTC TACTTTTAAT TCTTTAATAT 300

TATTTTTCAT TTCAAAAAGT GTGAAATAAA AATTAAAATG TCATAACAAT TTATTAACGA 360

CAAAATAAGA GAATAAGAAA AAAGAATACA AGCCAAATGA TATAATTTAT TTATATTATT 420

CATTATAGAC TTCATAATCT TCAGAGTTTA AAGCTACAGT ATACTCTGAA TAATTCTTTT 480

TCACAAAGAA ATACCAGTAG ACAGCACCAA CCGCCATAAC TGCTATTCCA CTAACTGGGA 540

AAACCCAATA TGCATAGCCG TCATTTTCCA CACTTCCATT TGGCGGTACG AATGGAACGA 600

CAGCCAAGAA AAGATTAGAG ACAATAAATA TTCCCGTCAA TACATACCAA CTTGTCCATG 660

GTGTCGGGAT ATCATTCCAA TTGTCATTAT TTGTCCAATG CATATATACC AACCCAACTC 720

CGATAATTAG ATTGAACCAA CCACTTGGAT ACGCGTAAAG ATCAATAATT AATTGATAAA 780

CGTTACCATT TGGTGGAATT ATGAGGACCA AAATAGTGAC AAAGCAGTGT AAAAATAGAG 840

CGTAGTTTAA TTTACTGAAA ATATGCGTAA AGGGGAAAAT TCCTTCTTTA GCAAGTTCTT 900

GGTTTACTCT GGCATTTGAG AAACTAACGG CCATAACGTT ACCATAATTT GAAAGTGTTA 960

TGCAGAAGGG TAAAAAGCGT GAAACAATCT GTGCCGCCAA AGATTTTGAG TATTTCTCAA 1020

AAATTTTCTC AAAGAAAACA CCACTTATGA GGATACCCGT ATTTGATATT TCATCTTTGG 1080

GGATTACAAT GTAGTACGAG ATCACTATTG CAATATATAG GAAAGTTGTA AGACCAACCG 1140

ATACTGGTGC TGCAATCATT AGAGTTTTAT GAGGATCCTC GAG 1183

***Kluyveromyces marxianus* DMKU3-1042 DNA, autonomously replicating sequence, *Km*ARS51**

Note="*Km*ARS51 (491-540); minimum sequence for autonomous replication"

GAATTCAGAT ATGACAATGA GAGGTACAGT TTTTTGGATG GCGCCAGAGA TGGTGGATAC 60

TGCCCATGGA TACAGTGCTA AAGTAGATAT ATGGTCTCTC GGATGTGTTG TATTGGAAAT 120

GTATGCTGGT AAGAGACCGT GGTCAAATTT CGAAGTTGTT GCTGCTATGT TCCAGATAGG 180

GAAATCTAAG ACTGCGCCTC CTATACCTGA CGATACAAAA GACTTAATAT CAGCAAGTGG 240

TAAAAACTTT TTAGACATGT GTTTCGAAAT AAACCCGGAG AAAAGACCTA CCGCTGATTT 300

CTTAGTGAGT CACCCCTTCT GTAAAACAGA TCCGTCATTC GATTTTTCTA AAACTAAACT 360

AGCCCAATTC ATCAGGTCAA ATGATAAACT AAACAACAGT AAGTTACGTA TCAGCTCACA 420

AGAGTTATGA AGCTTGGATT TATTGAGTGT CTTGTTATAT ACATACGTAT TATGCTCTAA 480

AACTTCTTTT AATATTTATG AATAAAAGTA ACTTTTTAGT TTCAAATACT AAAAAATATT 540

AATTACAAAG TAACAATATA ACCAATTATT ACAAAAAAAT GCCAGATACT ACACAAGGAA 600

TTACGTTTAT TAAGTATGAT CATGGGATTT CATTTCCCCA ATTTCGACAT CTTCTAAAAG 660

ATCATCTAAA ATTTCATTAA GCATGTCAGA AAGACGGTAT GTCAAATATA ATGATTGCTT 720

GAAAGGATAT CGGAGAGGAG ATTCCTCAGA AATCCAAAAG AGCTTTGATT GTGACTGGTT 780

TAAACGTTTC TCGAG

***Kluyveromyces marxianus* DMKU3-1042 DNA, autonomously replicating sequence, *Km*ARS14**

Note="*Km*ARS14 with autonomous replication sequence"

AATTCTCTCT CTCTCTCTCT CTTTTTCCGT TCCCATTTCC AAGGTTTGCC GTTTTTTGCG 60

GAATCCGAAT TGGAACCTGA ACCTGAACCT GAACCTGAAC CTGAATATGA AATCTGAGTC 120

TGAGTGAGTT CAAATCTGAA TGCAACTGCA ATCATCCGCA GGCGCACTTG ACAATACCCC 180

TCCCAACTTC ATTATATAGA TGTACTTTTT TAGGTCTATA AAAAATCTCC ATCCATTTCA 240

GTACATAGTA CATACTTTAT CTTTTGCGTC ATCGAAATAC TAGAAAATAA AAAAATACCA 300

TATCACACCT ATTTCCTCTT TTCTCTCTGT CCAAGCACAT TTGGTTATCG AAAGCTGTCA 360

ATGAGCTTAC AATTTGTAAT TACATATATG TATCACATAC ACAAGTACAC CAAAAAAAAA 420

GGCCCCACAA TAGCAGGGAA AAATATTTAC ATACACACAC ACTCTCGCAA CAACCCACCA 480

CGGCCCACCA GGGCCCGCCA CAACACCGGC AACAACCCTG TAACCGCCGC CTCTTTCCTT 540

CCTCCTCTCC CTCATCACTC TATACGTTTT GATCTCTCGA ATTTCCAGCT TAAAGCTTTG 600

AAAATATAAT GATTCTTTTA AACATAGGAA AAAAGTAGAA AAAACTAACA ACTTGGGCGC 660

ATGTAATAAA GAACACGAAA AAGACAACAA AATATAGCCA CTACAAGAGC CAAACACAGG 720

GGAAATAACG GAACTAACAG AGCCAATTGA AGCTCTAAAA TCTGAAATCT AAAGTTTCAA 780

ATCGGGAAAT TGTATTAGAG AGTTGATTTT CCGCCCTTTC TTTCGTGTAA AGGACTCCAG 840

ATTAGATAGA TGCTTACAGA TTATCAGCGG TCCGATGCCG CTTCCATTCT GCCCACGGTG 900

GAGAGATTAT TTGCTGAAGG AGTGTTTAAG AAATATTTGA ATCCGAGCCA ATTGCTTCTT 960

TTGAAGTCGC TACTGCACCT TCGTGATTCT GAATTGAAAG CGCGCATATG GGATTCATTC 1020

CTTACGGGGA AACTCGAG 1038

***Kluyveromyces marxianus* DMKU3-1042 DNA, autonomously replicating sequence, *Km*ARS45**

Note="*Km*ARS45 with autonomous replication sequence"

GAATTCCTCA GAATTAGAAG TACGTAGTAG AAGGTTTCTT GTCGCTATAC TCGTTTTAGT 60

AAGTTTTTTG TATGTGTATG TATTGTACTT AAGCGTAGGG CAGGACCCTT CAGGACGACA 120

ATATTGTCAA AAAATAAATA CAACAAAATG AGAAAGAGAA AAATATTTAA AGATTTAAGA 180

AGGAAAAAGA GAATTGGGAA AGGAAAGTGG AAATTGGGTT CTCGGCTGTT TTTCGATATA 240

CTATACTCGC ATGAGGGAAC AAGTTTCAGA CGATGGATAA CACGTGAGAT CATCATTTGT 300

GTATCCACCG GTGCATCTAG CGGCTTTCTA TTTGGCTTTG AAGTCGGATC TGACTGACAA 360

AAAAATAGTG CTAGAAAGCT ACTACAATCA GTTATAAGAT TTAGACGATA ATGAGATGTG 420

CAAAACCCTA GTGACACCAG TCTTCGGTGC TTTTCACATC AACCAACCGT CATCACTAAA 480

TTCAACCCAC TAAGTAGTTG CAAAAGATTT TAGATGGTGA AATGAAAGTG AGAATTTAGA 540

TAGGAAAGAT AGAGAATCCA TGCTCGAG 568

***Kluyveromyces marxianus* DMKU3-1042 DNA, autonomously replicating sequence, *Km*ARS3**

Note="*Km*ARS3 with autonomous replication sequence"

GAATTCGAGA ATGTGGATTC TGTTGCAAAG GCTTTGGAAA AGAAGGATTC TCAGCTACAT 60

GGCGAAACTA TCAATGTAGC TAAGAAAAGA ACTAATGTTC CTGGCTATAA CAATAAAAAC 120

AACTATCCAA GAAAGGAATG GATTTATCCC AGCTGGCAAG GAAATGTTGT TCGCCAGTGG 180

TATTATCCAT ACTCTGCACC AGCTTACCAA AACTATAAGC CAAATGGCAT GCCTATGCCA 240

CCACCTCCCC CTCAAAACGC ATTTTACTCT CAATACCCCT ACGTTTCCCG CCCAGCTCAC 300

AATGGCGGTC AGAAGATGCC AGGTTATTCC AGAGGATACT ACAGAGGTAA TTACAGAGGT 360

AATTACAACG GGAGAAGAGG TAACAGTAAT TATAGAGGAC CAAGGAATCA AAATAATGGC 420

CACTATTCTA GACCAGACTC TCACCGCAAT GAGAATGGTG ACGCTACCCC TGCTACTGCT 480

ACTGCCACTA CTCCTTCTCC TTCTACTACC TCTCCGGTTT CTGAAACTTC AGAAGGAAAC 540

ACACAAGTTG AGACAGTGGA AACTACCTCT GATGTACCAG CTTCAAAGGT TGAGGAATGA 600

TTTATACGAA ATATATTCAA TTCAATTACG TGAAAATAAT ATCCATTAAT GAAAAAAAAA 660

TGGAATAATG GCTCGTACAA GTTCCTTTTC CTTTTGTCTA ATTATTACGA CTTCAACTTT 720

CATGACGCTT CTCAATGGTT AATCAGTACA GCTCAATTAT CTGTAAATAG TGTTGTTTCC 780

TTTCGGCTTT TATCCACTCA TTGTGTTCTA CAACTTTGTT TTGTTTGTTT CATCCTTTTT 840

CTATGTTAAC ATTGAGAAAG CTTCAAGAGT TGTTTGTATT TATTCGAAAT ATGCACACCT 900

GATGGATTTC ACCAACATGA ATGTTTCATT CCAAAAAGGT AGGTTCAATG GTGCATATGT 960

GTACGTCATG TAACTCTTTT TGTAGTTTCT ACCCGCAGAA GTTAAGCATT TAACAATCTA 1020

TTCACCGCAA TGTTTGAAAA TATGTGACGA CAGTTATCTT AGGTAGTTGA GACAATTAAT 1080

GCACTAAATT TTTACTAAGG GAAATATAAC TTCATACACT ACAGATTATG TACCCAATTG 1140

GTGTTTTCAC ATATTATATA TGTAACACTA TAACTACAAC CATATACTTT TAAATATTCT 1200

TTTAATTGAT CTCCTTTCAT TTCTGATAAA AGTAAGGCTT CTCTATTTAC CTTTTAACCT 1260

ACATATTCAT AGTTGGAAGT TATCCTTCTA AGTACGTATA CAATATTAAT TCAACGTAAA 1320

AACAAAACTT ACTGTAAATA TGTGTAAAAA AAATCTATTA AATTCATGGC AGTTTCAAGA 1380

AAAGAAAACT ATTATGGTCT GGTCACGTGT ATACAAATTA TTAATTTTAA AACTATATAA 1440

TTTATTATTT TTTTATTTTG AAGTTTAGAG TAATTTTAGT AGTATTTTAT ATTTTAAATA 1500

AATATGCTTT AAATTTTTAC TTAATATTTT ATTATTTTTA AATACAACGT TTTTATTTAA 1560

AACAAAATTA TAAGTTAAAA AGTTGTTCCG AAAGTAAAAT ATATTTTATG GGTTTTACAA 1620

AAATAAATTA TTTTTAATGT ATTTTTTTAA TTATATTTTT GTATGTAATT ATATCCACAG 1680

GTATTATGTT GAATTTAGCT GTTTTAGTTT ACCTGTGTGG TACTATGATT TTTTTAGAAC 1740

TCTCCTCTTA GAAATAGGTG GTGTTGCGGT TGACTTTTAA CGATATATCA TTTTCAATTT 1800

ATTTATTTTA AAGTGACATA GAGAGATTCC TTTTAATTTT TTAATTTTTA TTTTCAATAA 1860

TTTTAAAAAT GGGGGACTTT TAAATTGGAA CAAAATGAAA AATATCTGTT ATACGTGCAA 1920

CTGAATTTTA CTGACCTTAA AGGACTATCT CGAACTTGGT TCGGAAATCC TTGAAATGAT 1980

TGATATTTTG GTGGATTTTC TCTGATTTTC AAACAAGTAG TATTTTATTT AATATTTATT 2040

ATATTTTTTA CATTTTTTTA TATTTTTTTA TTGTTTGGAA GGTAAAGCAA CAATTACTTT 2100

CAAAATATAT AAATCAAACT GAAATACTTA ATAAGAGACA AATAACATTC AAGAATCAAA 2160

TACTGGGTTA TTAATCAAAA GATCTCTCTA CATGCGCCCA AATTCACTAT TTAAATTTAC 2220

TATACCACTG ACAGAATATA TGAACCCAGA TTAAGTAGCC AGAGGCTCTT CCACTATATT 2280

GAGTATATAG CCTTACATAT TTTCTGCGCA TAATTTACTG ATGTAAAATA AACAAAAATA 2340

GTTAGTTTGT AGTTATGAAA AAAGGCTTTT GGAAAATGCG AAATACGTGT TATTTAAGGT 2400

TAATCAACAA AACGCATATC CATAGTGGAT AGTTGGATAA AACTTCAATT GATCCAGAAG 2460

CATCAAATAG CAACAAGAGA TCGGTATCAA CAATGAAGTT CTCAAAGATT CTTTTATTTT 2520

CCTCGAGTTT GACGGCTGCT TTTGCTCAAA TTCAGCAACA CATCGATGTT AGTGATATTA 2580

AGATAGATAC 2590

***Kluyveromyces marxianus* DMKU3-1042 DNA, autonomously replicating sequence, *Km*ARS20F**

Note="*Km*ARS20F with autonomous replication sequence"

TTTTTCAAAA TGGAGCAGGG AAAAAGTGTT ACGGATAAAA AAAGGCTCCA CTCTACCAAA 60

TAAACTTGAT ACAATTCTGA ATATAACAAG AGTAAAGTTA GTGCCAAACC AACCTTCAGA 120

CTTGGATCAT CCACAGATAA TCAAGATGCC AACAGTTCAA ACCCTAGCGG ATGTTTGTAT 180

GGTGCCTGTA TGTTAGAAAC AATCTAAGAA CTCTTTTAAT AGGATGAGTT GATTTCCTCT 240

CCCCTGTATT TGACCTTATG GCTTTCAGTT CAATTGTACT AACAATATGT GAAATTCAGT 300

GCTTACTGTA GATTTTATAC CTACAGTAAG CCGTTAAATT GACATTGCTA ATAGATTGGT 360

ACCGGATCAC CAAGTGTTTC CGATTTTGTT ACTAAGATCG AAAAGAAGAT CAGGGAATCT 420

CATTTGAAAA GCACTTTGCA TAGTGCTGGG ACGACTATTG AAGGCCCATG GGATGAAGTC 480

ATGAAATTAA TTGGTGAGCT TCATGAATAC AGCCATGAAC TAGGTTATGT TAGAGTTCAC 540

ACTGACATCA GAATCGGAAC ACGTTCTGAC AAGAAGCAAA CTGCTGAAGA TAAGGTTAAT 600

GCTGTTCTTT CCAAGCTTGA CAAATAAATG CACCCAATTC GCTGGTTGTC AGCTTACAAT 660

TTCTCTTTTA AGCAATATAC ACATATAGCC ATTTAATACT GAAATATATG ATTATATTAC 720

ATCTTTCGAT TTATTTAGTC TACATGGTAT ACTTTAAATG GGGGACCCAG AGGTATATTT 780

AATATTAAGG GTAAAAAGAA GTTCACACAC CCTTAATGTG TTTAGATATA ATGATACAAC 840

TTTCTGCAAT AGAAATAAGT TGGAGAAATG AATTCCAATA GTCAATAATG GAAACGATAG 900

AACAAG 906

***Kluyveromyces marxianus* DMKU3-1042 DNA, autonomously replicating sequence, *Km*ARS20R**

Note="*Km*ARS20R with autonomous replication sequence"

CTCGAGATCC AGTTGAATAT GGGGCATACG AAGAAACATA AGGCTGCAAT GAAATACCAT 60

TAGTTTGATA ATTGTGTCCT TGATATTGTT GCTGGTATGT CTTTGGTACA GGTGTAATAA 120

ATGAGGATCC CATCGGTAGT AATTGGGTAC TTGATGAATG GGAATGCAAA ATGTGTCTAT 180

TGTTCTCGTA GTGATCCAAG TGTTGGGGAT GGTTTCTATG GGTAATGGTT GAATCTGATA 240

TGTATGAACT GTCTGATCTT GGAGAAAGAG AATCTGATGA TGAATAAAGA TCATTTGGAT 300

GTGGATTTTG TTGGTTACGA TTGTGATGGC TACGATGATT CTGATTTACA TCTGGACCAG 360

CCATCATTTC AGCTTCCTTC ATCATACCTC TTGAAGGATT AGAATCGCGG AAAACGTTAC 420

GTTGTCTCTT TTTCACATTG GTTTTCCACC AATTTTTTAT TGAATTATCG CTTCTATTGG 480

TATTCAGCAG CTTAGCTATC AGTGTCCAGC GAAATCCATG TTTTGTTACC AAGCTTTTAA 540

TAGCTTCGCC CTCCTCCCTT GAAATTGGTG TTTTGTTTAA ATTCGGCCGC AGGTTTTGAT 600

GATATCTTTC ACGGCATTGT TTAGCAGTAC GGGTTTTCAG TTGGTGTGAT ATTTCTACCC 660

ACCGCGAAGC CCCATACACT TCTATCAGAC TTAACAGCAT CCTATCTTCT TCTGCGGACC 720

ACAATCCCTT GATGGTCTTC GCTACAGTTG GAGATCCTGT CGGTACTGTC TGTGTATCTT 780

TCTTCTGATT TGTTTTTGAA TTCAGCATAT TCGAGTCTTT ATTATTGTTT TCGGTAGATG 840

ATGAACCCAC AGTGCTTTTG GTTTCTATAT CACTTTTCGA CTCCAAATTT TTATCTTCCT 900

CTTGC 905

**Fig. S2**. *K.* *marxianus* autonomously replicating sequences (*Km*ARSs). Sequences highlighted in yellow color represent the minimum sequence with ARS activity.

**
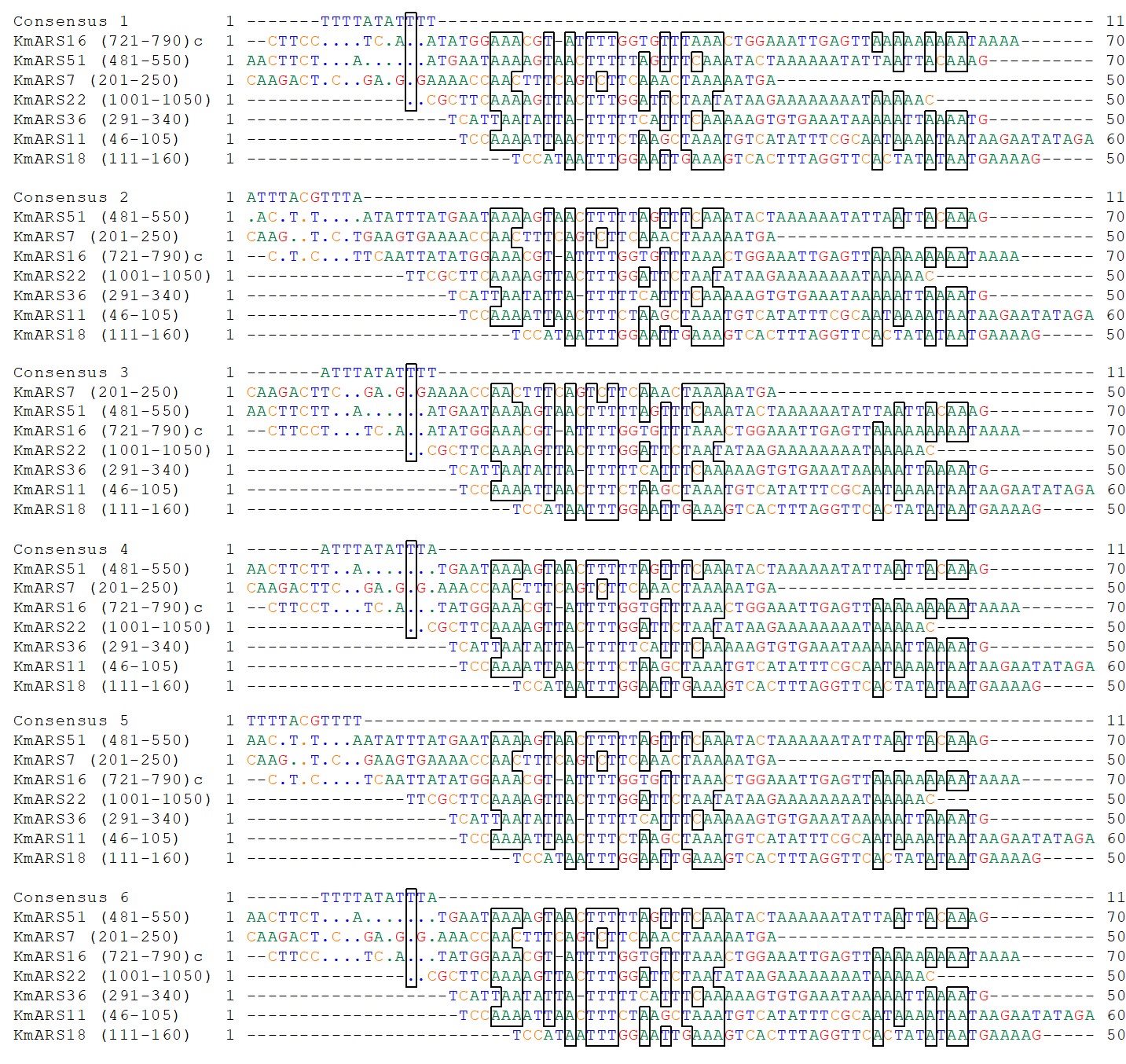
**

**Fig. S3.** Alignment of minimum active *Km*ARS sequences versus ARS consensus sequences of *S. cerevisiae*. Consensuses 1~6 represent those of *S. cerevisiae*.


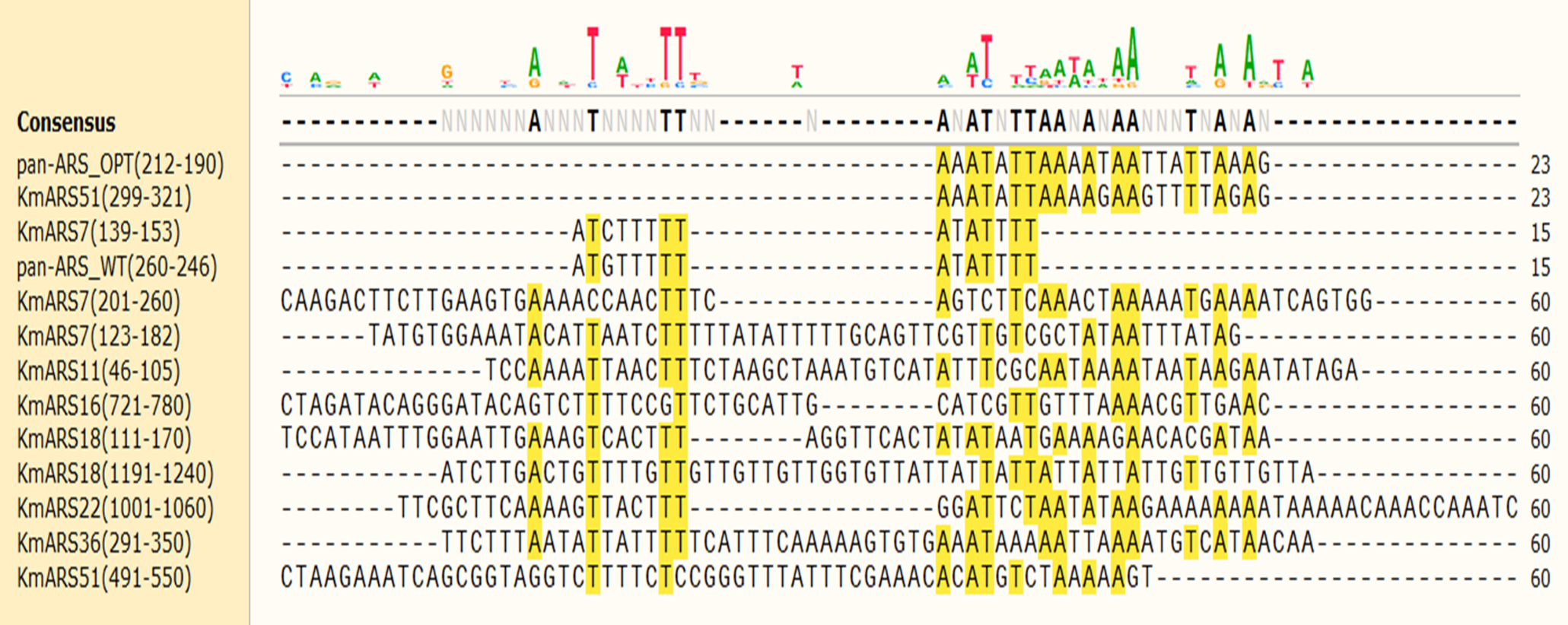


**Fig. S4.** Sequence alignment of *Km*ARSs (this study) and *K. lactis* ARSs consensus. pan-ARS OPT (212-190) is the optimized *Kl*ARS consensus and pan-ARS WT (260-246) is *Kl*ARS wild type consensus (Liachko and Dunham, 2014). The region *Km*ARS51 (299-321) with approximately 87% sequence identity to the pan-ARS OPT consensus. The region *Km*ARS7 (139-153) with 93% sequence identity to the panARS WT (260-246) consensus. However, the regions *Km*ARS7 (139-153) and *Km*ARS18 (1191-1240) have very low transformation efficiency relative to the regions *Km*ARS7 (210-250) and *Km*ARS18 (111-159), respectively (Suppl. Fig. 5).


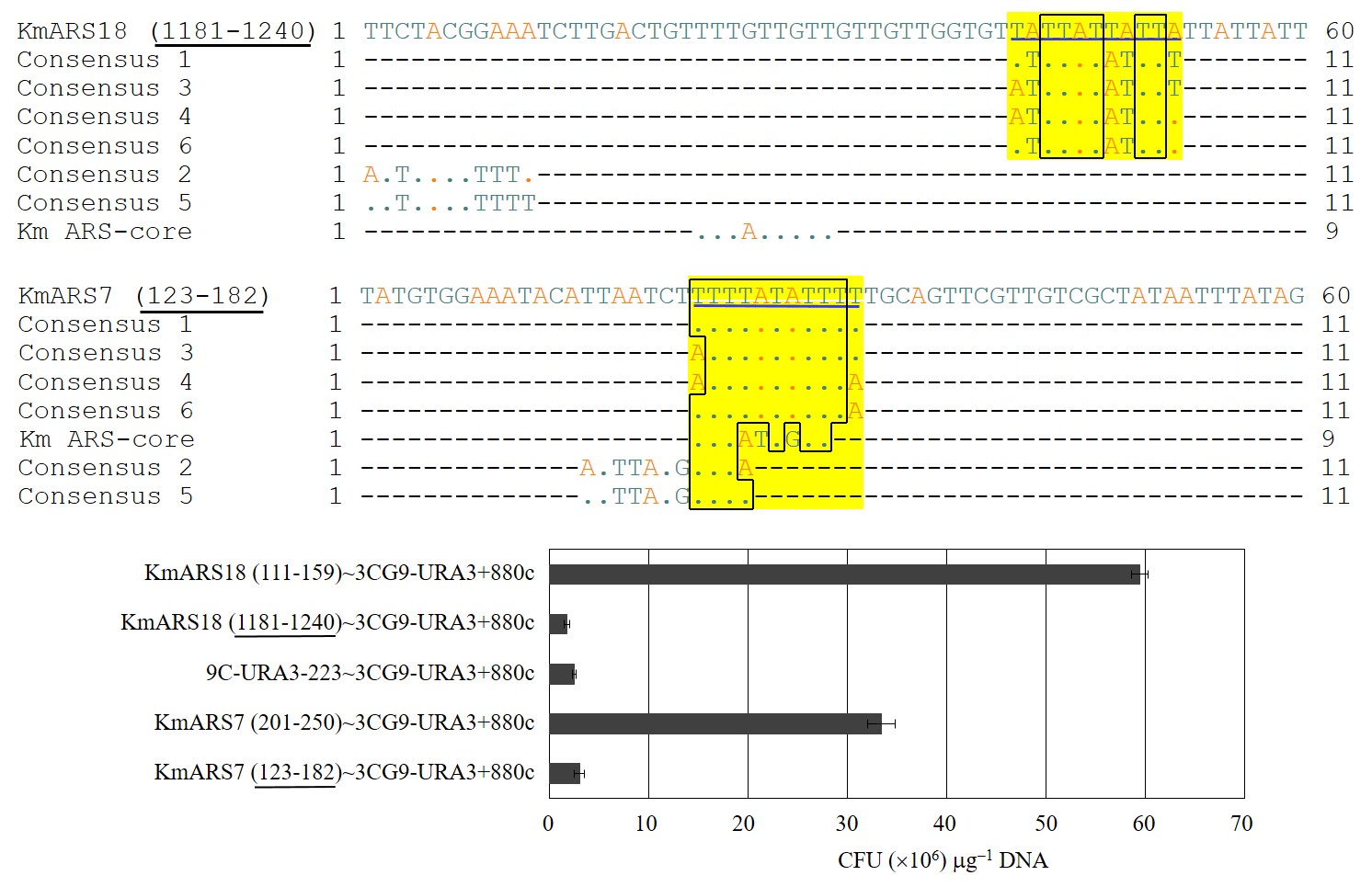
**Fig. S5**. Modular nature of *Km*ARS7 and *Km*ARS18. Sequences of *Km*ARS18 (1181-1240) and *Km*ARS7 (123-182) relatively with high identity to *K. marxianus* strain ATCC12424 ARS (Iborra and Ball, 1994) and *S. cerevisiae* (Deshpande and Newlon, 1992) ARS consensus (top panel), but functionally these regions are very weak compared to the regions of *Km*ARS18 (111-159) and *Km*ARS7 (201-250) (bottom panel).


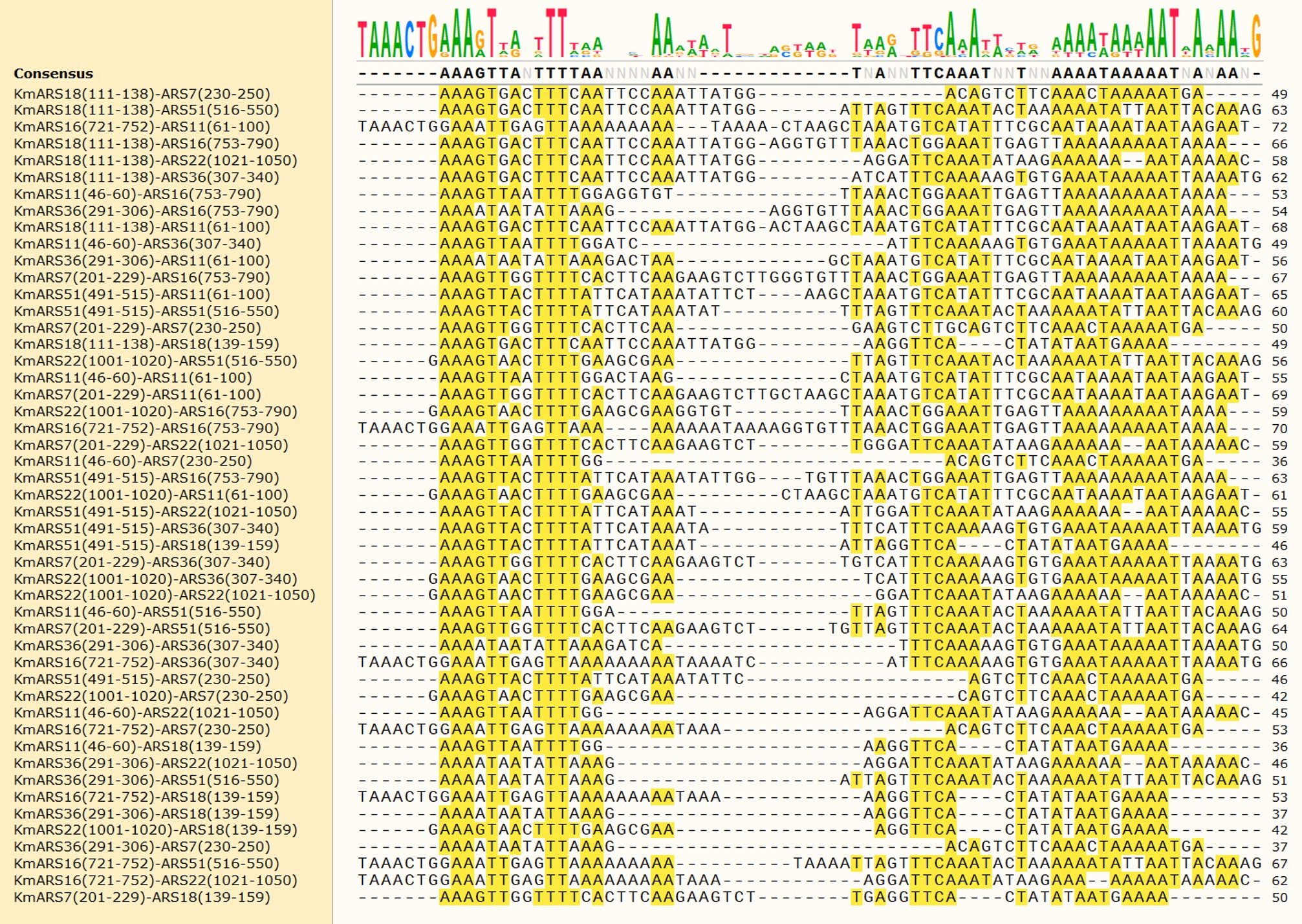


**Fig. S6.** Alignment of the interchanged sequences of *Km*ARSs showing the consensus with a threshold of more than 50 percent and a sequence logo for base composition.

**References:**

Deshpande, A.M., Newlon, C.S., 1992. The ARS consensus sequence is required for chromosomal origin function in *Saccharomyces cerevisiae*. Mol. Cell. Biol. 12, 4305-4313.

Iborra, F., Ball, M.M., 1994. *Kluyveromyces marxianus* small DNA fragments contain both autonomous replicative and centromeric elements that also function in *Kluyveromyces lactis*. Yeast 10, 1621–1629.

Liachko, I., Dunham, M.J., 2014. An autonomously replicating sequence for use in a wide range of budding yeasts. FEMS Yeast Res. 14, 364–367.
